# Dolutegravir dosing for children with HIV weighing less than 20 kg: pharmacokinetic and safety substudies nested in the open-label, multicentre, randomised, non-inferiority ODYSSEY trial

**DOI:** 10.1016/S2352-3018(21)00292-7

**Published:** 2022-02-18

**Authors:** Hylke Waalewijn, Man K Chan, Pauline D J Bollen, Hilda A Mujuru, Shafic Makumbi, Adeodata R Kekitiinwa, Elizabeth Kaudha, Tatiana Sarfati, Godfrey Musoro, Annet Nanduudu, Abbas Lugemwa, Pauline Amuge, Cecilia L Moore, Pablo Rojo, Carlo Giaquinto, Angela Colbers, Diana M Gibb, Deborah Ford, Anna Turkova, David M Burger, ODYSSEY Trial Team

**Affiliations:** a Department of Pharmacy, Radboud Institute for Health Sciences, Radboud University Medical Center, Nijmegen, Netherlands; b Medical Research Council Clinical Trials Unit at University College London, University College London, London, UK; c University of Zimbabwe Clinical Research Centre, Harare, Zimbabwe; d Joint Clinical Research Centre, Mbarara, Uganda; e Baylor College of Medicine Children's Foundation, Kampala, Uganda; f Joint Clinical Research Centre, Kampala, Uganda; g University Hospital October 12, Madrid, Spain; h Department for Woman's and Child's Health, University of Padova, Padova, Italy

## Abstract

**Background:**

Dolutegravir-based antiretroviral therapy is a preferred first-line treatment for adults and children living with HIV; however, very little pharmacokinetic data for dolutegravir use are available in young children. We therefore aimed to evaluate dolutegravir dosing and safety in children weighing 3 kg to less than 20 kg by assessing pharmacokinetic parameters and safety data in children taking dolutegravir within the ODYSSEY trial.

**Methods:**

We did pharmacokinetic substudies nested within the open-label, multicentre, randomised, non-inferiority ODYSSEY trial. We enrolled children from seven research centres in South Africa, Uganda, and Zimbabwe. Children weighing 3 kg to less than 14 kg received 5 mg dispersible tablets of dolutegravir according to WHO weight bands: 5 mg for children weighing 3 kg to less than 6 kg and younger than 6 months, 10 mg for children weighing 3 kg to less than 6 kg and aged 6 months or older, 15 mg for children weighing 6 kg to less than 10 kg, and 20 mg for children weighing 10 kg to less than 14 kg. Children weighing 14 kg to less than 20 kg received a 25 mg film-coated tablet once per day early in the trial or 25 mg dispersible tablets (five 5 mg tablets once per day) later in the trial. A minimum of eight children per weight band or dose was targeted for 24 h pharmacokinetic profiling at steady state. The primary pharmacokinetic parameter was the trough concentration 24 h after observed dolutegravir intake (C_trough_). Pharmacokinetic targets were based on adult dolutegravir C_trough_ and the 90% effective concentration (EC_90_; ie, 0·32 mg/L). Safety was evaluated in eligible children consenting to pharmacokinetic substudies.

**Findings:**

Between May 25, 2017, and Aug 15, 2019, we enrolled 72 children aged between 3 months and 11 years. 71 children were included in the safety population and 55 (76%) of 72 children contributed 65 evaluable pharmacokinetic profiles. Geometric mean C_trough_ in children on dispersible tablets in weight bands between 3 kg and less than 20 kg ranged between 0·53–0·87 mg/L, comparable to the adult geometric mean C_trough_ of 0·83 mg/L. Variability was high with coefficient of variation percentages ranging between 50% and 150% compared with 26% in adults. C_trough_ below EC_90_ was observed in four (31%) of 13 children weighing 6 kg to less than 10 kg taking 15 mg dispersible tablets, and four (21%) of 19 weighing 14 kg to less than 20 kg taking 25 mg film-coated tablets. The lowest geometric mean C_trough_ of 0·44 mg/L was observed in children weighing 14 kg to less than 20 kg on 25 mg film-coated tablets. Exposures were 1·7–2·0 times higher on 25 mg dispersible tablets versus 25 mg film-coated tablets. 19 (27%) of 71 children had 29 reportable grade 3 or higher adverse events (13 serious adverse events, including two deaths), none of which were related to dolutegravir.

**Interpretation:**

Weight-band dosing of paediatric dolutegravir dispersible tablets provides appropriate drug exposure in most children weighing 3 kg to less than 20 kg, with no safety signal. 25 mg film-coated tablets did not achieve pharmacokinetic parameters in children weighing 14 kg to less than 20 kg, which were comparable to adults, suggesting dosing with dispersible tablets is preferable or a higher film-coated tablet dose is required.

**Funding:**

Paediatric European Network for Treatment of AIDS Foundation, ViiV Healthcare, and UK Medical Research Council.

## Introduction

In 2020, approximately 1·7 million children younger than 15 years were estimated to be living with HIV, with the majority living in low-income and middle-income countries.^1^ These children need lifelong treatment with antiretroviral drugs to prevent HIV-related morbidity and mortality. However, globally, only 53% of children were receiving antiretroviral therapy (ART) at the end of 2019, and available age-appropriate formulations, particularly for young children, are often suboptimal, contributing to children having consistently worse treatment outcomes than adults.^2^

**Research in context**

**Evidence before this study**

Dolutegravir-based antiretroviral therapy (ART) is being used in adults globally as it offers better tolerability, fewer adverse drug reactions, fewer drug-drug interactions, and higher genetic barrier to resistance than other available ART options. Dolutegravir is low cost and therefore appropriate for low-income and middle-income countries. Currently, updated WHO guidelines recommend dolutegravir-based ART as the preferred first-line regimen for adults and children living with HIV initiating ART; however, no suitable paediatric dose and formulation was available for children unable to swallow tablets when the ODYSSEY trial started and the suggested dose by the European Medicines Agency for children weighing 15 kg to less than 20 kg given as film-coated tablets was based on very limited data. We searched PubMed for clinical trials and pharmacokinetic or cohort studies of dolutegravir in paediatric populations using the following criteria: “(pediatric[Title/Abstract] OR paediatric[Title/Abstract] OR children[Title/Abstract] OR infant[Title/Abstract]) AND dolutegravir[Title/Abstract]” up to Sept 15, 2021. This search produced four peer-reviewed papers: two papers from the ODYSSEY trial and two from the IMPAACT P1093 trial. ODYSSEY, the ongoing randomised controlled trial comparing dolutegravir-based triple ART with standard of care in children aged 4 weeks to 18 years, has previously published the trial design and the results of a nested dolutegravir pharmacokinetic study in children weighing 20 kg or more. The second paper showed that adult dolutegravir tablets provide appropriate pharmacokinetic profiles with no safety signal, allowing rapid roll out of adult dolutegravir tablets for children weighing 20 kg and more worldwide. IMPAACT P1093, the ongoing phase 1–2 paediatric dose-finding trial, uses an age-staggered approach to investigate pharmacokinetics and safety of dolutegravir formulations in children aged 4 weeks to less than 18 years. The published papers reported adequate dolutegravir exposures on the studied doses and good medium-term safety for treatment-experienced children aged 12–18 years. In addition, IMPAACT P1093 has presented abstracts describing pharmacokinetics and safety of dolutegravir film-coated tablets in children aged 6 years to less than 12 years and dispersible formulations in children aged 4 weeks to less than 6 years. Pharmacokinetic data for dispersible tablets in the younger children were supportive of dosing tested within the ODYSSEY trial, but there were small numbers of children and safety data were limited to 24 weeks.

**Added value of this study**

This ODYSSEY pharmacokinetic and safety study addressed dosing of dolutegravir using WHO weight bands in children weighing 3 kg to less than 20 kg aged 4 weeks and above. Dispersible formulations were shown to provide appropriate drug exposures with no safety signal. In collaboration with IMPAACT P1093, the ODYSSEY study accelerated dose finding for young children and provided sufficient data for timely licensing approvals of paediatric dispersible tablets. In addition, pharmacokinetic data from this study showed that average dolutegravir exposures on the 25 mg film-coated tablet dose in children weighing 14 kg to less than 20 kg—a dose higher than licenced by some stringent regulatory agencies at the time of the study—are lower than adult reference values and individual exposures in some children are lower than the 90% effective concentration target. The study showed that dosing with 25 mg dispersible tablets in children weighing 14 kg to less than 20 kg provides adequate pharmacokinetic profiles with no safety signal. In the absence of dispersible tablets, these results support treating children weighing 14 kg to less than 20kg with 40 mg film-coated tablets.

**Implications of all the available evidence**

The results of ODYSSEY and P1093 pharmacokinetic studies informed recently updated WHO 2021 guidelines on dolutegravir dosing for children weighing less than 20 kg. Together with the previous ODYSSEY published pharmacokinetic study, practical dosing is possible to achieve using only two formulations across all weight bands in children: 5 mg dispersible tablets in children weighing less than 20 kg and adult 50 mg film-coated tablets in children weighing 20 kg or more. This dosing will allow an individual child to continue on dolutegravir-based ART from early infancy through childhood to adulthood. Additionally, it allows harmonisation of treatment between children and adults at a programme level, which makes drug procurement much easier in countries. A recent agreement between Unitaid, Clinton Health Access Initiative, and paediatric drug manufacturers of the dispersible dolutegravir formulation allowed prices to be reduced for this formulation by 75%, making it affordable for low-income and middle-income countries. Overall, the study results will facilitate the roll out of dolutegravir formulations for children, which should contribute to closing the gap in treatment outcomes between children and adults.

Current WHO-recommended treatment for young children consists of two nucleoside reverse-transcriptase inhibitors combined with an anchor drug from a different class. The preferred anchor ritonavir-boosted lopinavir has very poor palatability and a relatively high rate of gastrointestinal intolerance making adherence difficult; it also requires administration twice per day and has numerous drug interactions. Raltegravir twice per day is recommended as an alternative anchor agent for children; however, this drug has a low threshold to resistance and therefore might compromise future options with second-generation integrase inhibitors.^3^ The more recently introduced integrase inhibitor, dolutegravir, has the potential to change the use of ART for children; in adults and older children, dolutegravir has high virological efficacy, good tolerability, minimal drug-drug interactions, and a high barrier to resistance.^4^, ^5^ These characteristics combined with a low effective dose and, consequently, low manufacturing costs and small formulation size have made dolutegravir a preferred treatment option for most countries regardless of income status.

Soon after the ODYSSEY trial started, the European Medicines Agency (EMA) and stringent regulatory authorities from Canada and Australia approved dolutegravir 20 mg given as two 10 mg film-coated tablets for children weighing 15 kg to less than 20 kg; however, this dosing was based on very limited data and no licensed doses for children weighing less than 15 kg existed. To address the dolutegravir dosing and the gap in safety data for children weighing 3 kg to less than 20 kg, we did nested pharmacokinetic substudies in the ongoing ODYSSEY trial, with dosing according to WHO weight bands.

## Methods

### Study design and participants

ODYSSEY (NCT02259127) is an open-label, multicentre, randomised, non-inferiority trial evaluating the safety and efficacy of dolutegravir plus two nucleoside or nucleotide reverse transcriptase inhibitors versus standard-of-care in children living with HIV (aged 4 weeks to <18 years) starting first-line or second-line ART. This Article reports the results of the intensive pharmacokinetic substudies nested within ODYSSEY, which enrolled children weighing 3 kg to less than 20 kg, from seven sites in South Africa, Uganda, and Zimbabwe (Durban International Clinical Research Site, Durban, South Africa; Chris Hani Baragwanath Academic Hospital Perinatal HIV Research Unit, Soweto, South Africa; Klerksdorp-Tshepong Hospital Complex, Klerksdorp, South Africa; Joint Clinical Research Centre, Mbarara, Uganda; Joint Clinical Research Centre, Kampala, Uganda; Baylor College of Medicine Children's Foundation, Kampala, Uganda; and University of Zimbabwe Clinical Research Centre, Harare, Zimbabwe). The main trial and substudies were approved by local ethics committees. Results of other nested pharmacokinetic substudies are reported elsewhere.^5^, ^6^

In the first substudy, we enrolled children weighing 14 kg to less than 20 kg. Following a review of results, we did a second substudy in this weight band, using a different dolutegravir formulation and dose. Participants in the first substudy were asked to reconsent to participate in the second substudy if they remained in the same weight band, with additional participants recruited into the second substudy. Children weighing 3 kg to less than 14 kg were only enrolled in ODYSSEY if their parents or guardians consented to participation in the pharmacokinetic substudy and if they were randomly assigned to receive dolutegravir, because at this time dolutegravir doses were not approved for these weight bands. Children with illnesses that could affect pharmacokinetic results, including severe diarrhoea, vomiting, renal or liver diseases, and severe malnourishment; and those on concomitant medication known to have drug-drug interactions with dolutegravir were not eligible for inclusion in the pharmacokinetic substudies.

### Procedures

Dolutegravir dosing was once daily. In the first substudy children weighing 14 kg to less than 20 kg were treated with one 25 mg film-coated tablet instead of the licenced two 10 mg film-coated tablets of dolutegravir, which aimed to simplify dosing and reduce the number of formulations needed for resource-limited settings. In the second substudy, children weighing 14 kg to less than 20 kg were treated with a 25 mg dose, given as five 5 mg dispersible tablets, which is known to have higher bioavailability than the equivalent film-coated tablet.^7^, ^8^ Children weighing 3 kg to less than 14 kg were given 5 mg dispersible tablets that resulted in the following doses according to WHO weight bands: 5 mg for children weighing 3 kg to less than 6 kg and younger than 6 months, 10 mg for children weighing 3 kg to less than 6 kg and aged 6 months or older, 15 mg for children weighing 6 kg to less than 10 kg, and 20 mg for children weighing 10 kg to less than 14 kg.

Children in the pharmacokinetic substudies visited the clinic at 0, 2, 4, and 12 weeks, and then once every 12 weeks after starting dolutegravir or changing to dispersible tablets from film-coated tablets. At each visit, we did a clinical assessment and measured haematological and biochemical variables. We ascertained serious adverse events (defined according to the International Conference on Harmonisation Guideline for Good Clinical Practice), grade 3 or higher clinical and laboratory events, and events of any grade resulting in modification of ART.

Children were on the dolutegravir study dose for at least 7 days to achieve steady-state before the 24-h pharmacokinetic profile. We took blood samples at baseline and 1, 2, 3, 4, 6, and 24 h after observed dolutegravir intake. Blood sample volumes were within blood draw limits for children established for research studies.^9^ For children weighing 10 kg or more, 3 h fasting before dolutegravir dose and 2 h after the dose was required. For children weighing less than 10 kg, fasting was preferred for 2 h before and 1 h after; more leniency was allowed to make it easier for young children for whom adhering to strict food scheduling is difficult. Drug-intake details as well as sampling procedures and full fasting requirements are available in the appendix (p 2). Intake of co-medications other than antiretroviral drugs was not allowed within the first 2 h after dolutegravir.

Blood samples were refrigerated within 10 min and centrifuged within 24 h after collection. Plasma was separated and stored at –80°C until shipping to the core laboratory site for quantification. We measured dolutegravir plasma concentrations with a validated liquid chromatography mass spectrometry bioanalytical quantification method with the lower limit of quantification of 0·01 mg/L.^10^

### Outcomes

The primary pharmacokinetic parameter was the trough concentration 24 h after observed dolutegravir intake (C_trough_). Other pharmacokinetic parameters measured were area under the concentration-time curve over the dosing interval (AUC_0–24 h_), maximum plasma concentration (C_max_), baseline concentration (C_0_), apparent elimination half-life (T_1/2_), oral clearance (L/h), and distribution volume (L). Pharmacokinetic targets were based on adult dolutegravir C_trough_ and the EC_90_ (concentration at which 90% of the maximal viral load reduction was obtained in a 10-day monotherapy study) of 0·32 mg/L.

### Statistical analysis

We aimed to enrol at least eight children with evaluable pharmacokinetic results per weight band and formulation. 80% power is reached with eight individuals and a coefficient of variation of 31–35%.^11^ The coefficient of variation percentage of our primary pharmacokinetic reference parameter (ie, C_trough_) was 26% in adults, so eight participants would give the analysis sufficient power at equal coefficient of variation percentage.^12^

We considered a pharmacokinetic curve non-evaluable if more than one of seven blood samples haemolysed or if a participant was considered to be non-adherent on the basis of measured dolutegravir concentrations (predefined as C_trough_ being >15 times higher than C_0_), if protocol violations had occurred or if concomitant medication was used that could interfere with dolutegravir pharmacokinetics. We used Phoenix 64 software (version 8.1) for non-compartmental analysis to determine pharmacokinetic parameters for dolutegravir (AUC_0–24 h_, C_max_, C_trough_, C_0_, T_1/2_, oral clearance, and distribution volume). The AUC_0–24 h_ was calculated by a linear-up and log-down trapezoidal method.

The aim was to achieve pharmacokinetic parameters comparable to published values of approved adult dolutegravir dosing. In particular, we aimed for similar geometric mean C_trough_ to adult geometric mean C_trough_ on 50 mg of dolutegravir taken once a day under fasted conditions, and compared geometric mean AUC_0–24 h_ and C_max_ to geometric mean adult reference values for 50 mg of dolutegravir taken twice a day representing high dolutegravir exposure with a good safety profile.^12^, ^13^, ^14^ C_trough_ was chosen as the main pharmacokinetic parameter because it is considered to correlate best with virological response.^12^, ^15^ The concentration at which 90% of the maximal viral load reduction was achieved in a 10-day monotherapy study (EC_90_; 0·32 mg/L, calculated from data described by Min and colleagues^12^ and confirmed in the Spring-1 study^16^) was chosen as the target C_trough_ for individual participants. The percentage of children with C_trough_ below the target concentration was reported. The in-vitro 90% inhibitory concentration (IC_90_; 0·064 mg/L) for dolutegravir was used as an absolute minimum concentration for reference.^17^

To assess the effect of formulation in children weighing 14 kg to less than 20 kg, we did a mixed-model bioequivalence analysis with formulation as a fixed effect and subject as random effect for children taking film-coated tablets or dispersible tablets. Apparent clearance, C_trough_, C_max_, and AUC_0–24 h_ were compared between the two formulation groups—reporting geometric mean ratios between the pharmacokinetic parameters of both groups—and to geometric mean ratios from a study in adults comparing the same dose and formulation.^15^, ^18^

A descriptive analysis of C_trough_ (above or below EC_90_) and virological outcome at week 48 was done in children who started and completed pharmacokinetic profiles on dispersible dolutegravir formulation. If a week 48 viral load was not available, the next viral load after week 48 was used. The safety population included all children consenting to inclusion in a pharmacokinetic substudy excluding those with major ineligibilities. These children contributed safety data from the start of their examined pharmacokinetic dose for 24 weeks. An independent endpoint review committee who was masked to treatment allocation reviewed all reported adverse events. We did a descriptive analysis of follow-up and adverse events by dose and weight band using Stata (version 16.1). The association between log AUC_0–24 h_ or log C_max_ and occurrence of adverse events (binary outcome: yes or no) was examined by fitting logistic regression models (unadjusted and adjusted for weight bands).

### Role of the funding source

ViiV Healthcare reviewed and provided comments on the manuscript. Employees of UK Medical Research Council and Paediatric European Network for Treatment of AIDS Foundation were authors of the paper who were involved in study design, data collection, data analysis, data interpretation, or writing of the report.

## Results

Between May 25, 2017, and Aug 15, 2019, we enrolled 72 children from South Africa, Uganda, and Zimbabwe; of whom, 71 were included in the safety population, all aged between 3 months and 11 years. One child was excluded because of major ineligibility: enrolled as ART naive while taking ART supplied by an alternative clinic. One child was mixed Black-White, all others were Black African; 41 (58%) of 71 were female. All children weighing 14 kg to less than 20 kg entering the second substudy (25 mg dispersible dolutegravir tablets) had been previously exposed to 20 mg or 25 mg film-coated tablets of dolutegravir within the trial, with median previous exposure of 29·6 weeks (IQR 23·0–37·3; table 1).

**Table 1:** Safety population patient demographics and characteristics by weight band at time of starting the examined pharmacokinetic dose (per-protocol doses)

		**3 kg to <6 kg (<6 months)**	**3 kg to <6 kg (≥6 months)**	**6 kg to <10 kg**	**10 kg to <14kg**	**14 kg to <20 kg**	**14 kg to <20 kg**
Dose		5 mg	10 mg	15 mg	20 mg	25 mg	25 mg
Formulation		DT	DT	DT	DT	FCT	DT
Participants consenting to pharmacokinetics		8	1	20*	12*	24†	18†
Sex							
	Male	4 (50%)	0	6 (30%)	5 (42%)	13 (54%)	8 (44%)
	Female	4 (50%)	1 (100%)	14 (70%)	7 (58%)	11 (46%)	10 (56%)
Ethnicity							
	Black African	8 (100%)	1 (100%)	20 (100%)	11 (92%)	24 (100%)	18 (100%)
	Mixed Black-White	0	0	0	1 (8%)	0	0
Age, years							
	Median	0·3 (0·3 to 0·4)	1·3‡	1·3 (0·9 to 2·0)	2·6 (2·0 to 3·3)	6·2 (5·1 to 7·4)	5·8 (5·2 to 7·1)
	Range	0·3 to 0·5	..	0·4 to 2·9	1·8 to 5·9	4·0 to 10·8	3·6 to 8·5
Weight, kg							
	Median	4·8 (4·3 to 5·0)	5·6‡	8·1 (6·5 to 8·9)	11·3 (10·3 to 12·3)	17·1 (15·2 to 18·1)	17·9 (16·0 to 18·7)
	Range	4·1 to 5·4	..	6·0 to 9·9	10·0 to 13·0	14·2 to 19·5	14·2 to 19·3
Weight-for-age§							
	Median	−3·2 (−3·6 to −2·4)	−4·5‡	−2·3 (−3·3 to −1·0)	−1·6 (−1·9 to −1·1)	−1·6 (−2·5 to −0·7)	−1·3 (−1·8 to −0·5)
	Less than −3	4 (50%)	1 (100%)	6 (30%)	1 (8%)	2/23 (9%)	0
	−3 to less than −2	2 (25%)	0	5 (25%)	1 (8%)	6/23 (26%)	3 (17%)
	−2 to less than 0	2 (25%)	0	8 (40%)	8 (67%)	15/23 (65%)	14 (78%)
	0 or more	0	0	1 (5%)	2 (17%)	0	1 (6%)
	Missing	0	0	0	0	1	0
BMI, kg/m^2^							
	Median	14·7 (13·6 to 15·4)	13·7‡	15·0 (14·0 to 16·5)	15·3 (14·4 to 16·0)	14·4 (13·9 to 15·4)	14·8 (14·0 to 15·8)
	Range	13·1 to 16·5	..	12·3 to 19·5	13·1 to 18·3	13·3 to 16·3	13·5 to 16·9
BMI-for-age§							
	Median	−1·8 (−2·5 to −1·1)	−1·9‡	−0·9 (−1·7 to 0·4)	−0·2 (−1·2 to 0·4)	−0·7 (−1·3 to 0·1)	−0·4 (−1·0 to 0·3)
	Less than −3	0	0	1 (5%)	0	0	0
	−3 to less than −2	4 (50%)	0	3 (15%)	0	0	0
	−2 to less than 0	4 (50%)	1 (100%)	9 (45%)	7 (58%)	17 (71%)	10 (56%)
	0 or more	0	0	7 (35%)	5 (42%)	7 (29%)	8 (44%)
Previous ART							
	Receiving first line	8 (100%)	1 (100%)	17 (85%)	7 (58%)	7 (29%)	8 (44%)
	Receiving second line	0	0	3 (15%)	5 (42%)	17 (71%)	10 (56%)
Dolutegravir exposure, weeks							
	Any exposure	2¶	0	0	1	0	18
	Median	0·0 (0·0 to 0·6)	0·0‡	0·0 (0·0 to 0·0)	0·0 (0·0 to 0·0)	0·0 (0·0 to 0·0)	29·6 (23·0 to 37·3)

Data are n, n (%), or median (IQR), unless otherwise stated. The safety population of 71 children also included two Black African children who were exposed to non-per-protocol starting doses: one child (male weighing 3 kg to <6 kg and younger than 6 months) was exposed to and only completed pharmacokinetic profile on 10 mg of dispersible tablets and another child (female weighing 3 kg to <6 kg and younger than 6 months) was exposed to 10 mg of dispersible tablets but did not complete pharmacokinetic profile because of death by a traumatic accident. ART=antiretroviral therapy. BMI=body-mass index. DT=dispersible tablet. FCT=film coated tablet.

* One child was exposed to and completed pharmacokinetic profiles on 15 mg of dispersible tablets while weighing 6 kg to less than 10 kg (12 weeks exposure) and 20 mg while weighing 10 kg to less than 14 kg; this child is included in both groups.

† 13 children participated in the first (25 mg film-coated tablet) and second (25 mg dispersible tablet) pharmacokinetic substudies (14 kg to <20 kg).

‡ IQRs and range not presented, as only one child weighed between 3 kg and less than 6 kg and aged 6 months or older starting 10 mg of dispersible tablet.

§ WHO Child Growth Charts and WHO Reference 2007 Charts. Weight-for-age calculated for those younger than 10 years and BMI-for-age calculated for those younger than 19 years. If children were 10 years or older, weight-for-age will be missing.

¶ Two participants were exposed to an incorrect dispersible dose of 10 mg while younger than 6 months for 9 days and 24 days, respectively.

**Figure 1:**
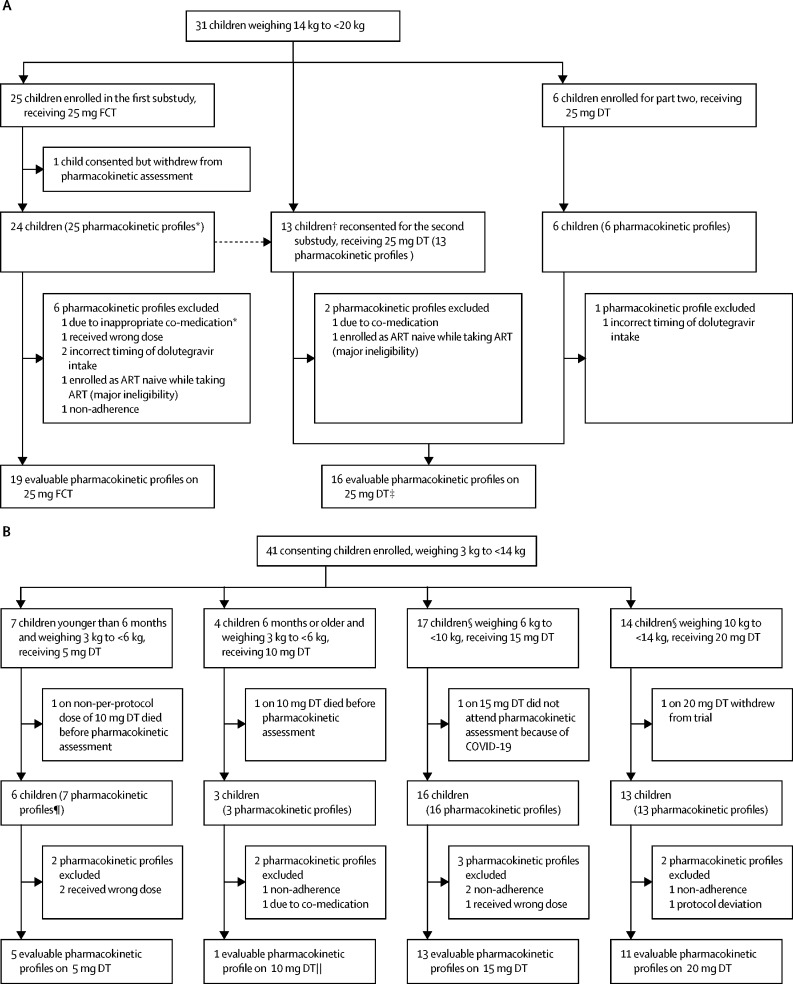
Substudy trial profiles (A) Participants weighing 14 kg to less than 20 kg. (B) Participants weighing 3 kg to less than 14 kg. ART=antiretroviral therapy. DT=dispersible tablet. FCT=film-coated tablet. *One participant weighing 14 kg to less than 20 kg did a pharmacokinetic assessment on 25 mg FCT while taking co-medication was excluded but subsequently returned for a pharmacokinetic assessment on 25 mg FCT without co-medication. †Including two participants who had been excluded from the first substudy because of co-medication or wrong dose. ‡Nine children had intrasubject comparison in the first substudy. §One child completed pharmacokinetic profiles in two weight bands (6 kg to <10 kg receiving 15 mg DT, and 10 kg to <14 kg receiving 20 mg DT). ¶One participant younger than 6 months weighing 3 kg to less than 6 kg did a pharmacokinetic assessment on the wrong dose (10 mg DT) and was excluded. This participant subsequently returned for a pharmacokinetic assessment at the correct dose of 5 mg DT. ||Results for this one child are presented in the appendix (p 3).

**Figure 2:**
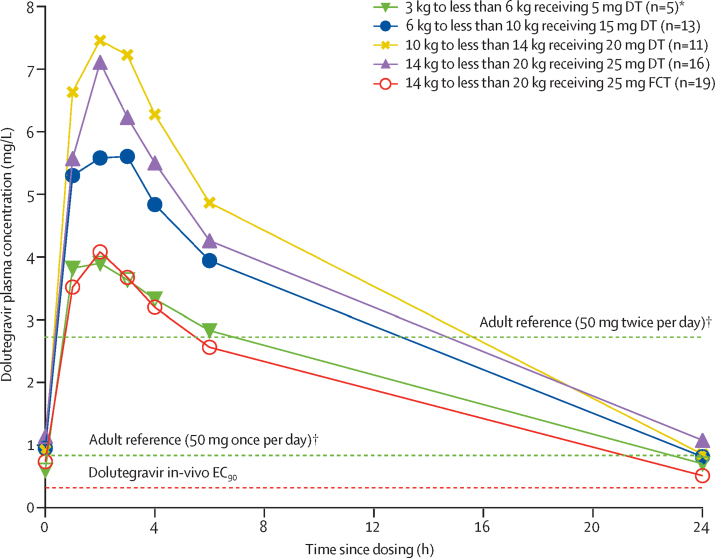
Mean plasma concentrations versus time profiles for all doses by weight band and formulation DT=dispersible tablet. EC_90_=the effective concentration at which 90% of maximal viral inhibition is achieved in a 10-day monotherapy study. FCT=film-coated tablet. *Children younger than 6 months. †Published geometric mean trough concentrations of adult reference values for 50 mg once per day and twice per day.

Of the 72 children enrolled, 55 (76%) children contributed 65 evaluable pharmacokinetic curves (figure 1). In total, 18 pharmacokinetic curves were excluded from the analysis (appendix p 5). Only one child older than 6 months in the 3 kg to less than 6 kg weight band had an eligible pharmacokinetic curve (appendix p 3). Table 2, figure 2, and the appendix (p 4) summarise the pharmacokinetic parameters and pharmacokinetic profiles by weight band, dose, and formulation.

**Table 2:** Summary of dolutegravir pharmacokinetic parameters by weight band and published adult reference values

	**3 kg to <6 kg (<6 months)**	**6 kg to <10 kg**	**10 kg to <14 kg**	**14 kg to <20 kg**	**14 kg to <20 kg**	**Adult (≥40 kg)**^12^	**Adult (≥40 kg)**^13^, ^14^
Dose	5 mg	15 mg	20 mg	25 mg	25 mg	50 mg (once daily)	50 mg (twice daily)
Formulation	DT	DT	DT	DT	FCT	FCT	FCT
Number of participants with pharmacokinetic profiles	5	13	11	16*	19*	10†	24‡
Age on pharmacokinetic assessment day (years)	0·3 (0·3–0·4)	1·4 (1·1–2·0)	2·7 (2·0–3·3)	6·0 (5·2–6·9)	6·2 (5·1–7·4)	..	..
Weight on pharmacokinetic assessment day (kg)	5·3 (4·9–5·3)	8·4 (7·1–9·6)	10·9 (10·3–12·0)	17·9 (15·5–18·6)	17·0 (16·0–18·6)	..	..
Dose (mg/kg)	0·9 (0·9–1·0)	1·8 (1·6–2·1)	1·8 (1·7–1·9)	1·4 (1·3–1·6)	1·5 (1·3–1·6)	..	..
Previous dolutegravir exposure on pharmacokinetic assessment day (weeks)	2·1 (2·1–4·0)	4·0 (3·0–5·9)	2·1 (2·0–17·9)	30·9 (25·9–41·3)	2·0 (1·9–3·4)	..	..
C_0_ (mg/L)	0·52 (75%)	0·46 (302%)	0·79 (59%)	0·98 (52%)	0·60 (74%)	..	3·20 (69%)
C_trough_ (mg/L)	0·64 (51%)	0·53 (150%)	0·77 (57%)	0·87 (64%)	0·44 (50%)	0·83 (26%)	2·72 (70%)
Number of participants below EC_90_	0	4/13 (31%)	0	0	4/19 (21%)	..	..
AUC_0–24 h_ (h × mg/L)	45·20 (33%)	52·23 (72%)	76·10 (21%)	69·93 (28%)	39·57 (32%)	43·4 (20%)	93·4 (50%)§
C_max_ (mg/L)	4·00 (32%)	5·61 (47%)	8·06 (21%)	7·20 (19%)	4·03 (31%)	3·34 (16%)	5·41 (40%)
T_max_ (h)	2·0 (1·0–2·0)	2·0 (1·0–2·2)	2·0 (2·0–2·3)	2·0 (2·0–2·0)	2·0 (2·0–2·0)	2·0 (1·0–4·0)	2·0 (0–7·9)
T_1/2_ (h)	8·79 (22%)	6·98 (29%)	6·95 (27%)	8·00 (32%)	7·28 (21%)	12·0 (22%)	..
Oral clearance (L/h)	0·11 (33%)	0·29 (72%)	0·26 (21%)	0·36 (28%)	0·63 (32%)	1·15 (20%)¶	0·54 (70%)¶
Volume of distribution (L)	1·40 (34%)	2·89 (43%)	2·63 (25%)	4·13 (19%)	6·64 (34%)	..	..

Data are n, geometric mean (coefficient of variation percentage), median (IQR), or n/N (%), unless otherwise specified. None were below IC_90_ 0·064 mg/L. Only one child who was 6 months or older in the 3 kg to less than 6 kg weight band receiving 10 mg dispersible tablet had an eligible pharmacokinetic curve (appendix p 3). AUC_0–24h_=area under the concentration-time curve from 0 to 24 h. DT=dispersible tablet. FCT=film-coated tablet. C_0_=baseline concentration. C_max_=maximum plasma concentration. C_trough_=trough concentration. EC_90_=the effective concentration at which 90% of maximal viral inhibition is achieved in a 10-day monotherapy study. IC_90_=90% inhibitory concentration. T_1/2_=apparent elimination half-life. T_max_=time to maximum plasma concentration.

* Nine participants had pharmacokinetic profiles on both film-coated tablets and dispersible tablets.

† Fasted adults who were HIV positive.

‡ Adults who were HIV positive and had previous treatment, fed state was not specified.

§ Calculated by doubling AUC_0–12 h_.

¶ Calculated by dividing dose over AUC_0–24 h_.

**Figure 3:**
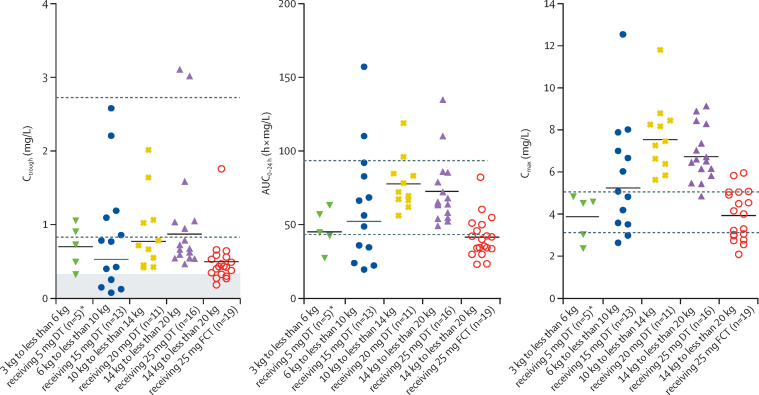
Individual dolutegravir C_trough_, AUC_0–24 h_, and C_max_ in children weighing 3 kg to less than 20 kg taking DT or FCT The horizontal black lines indicate geometric means per dose. The shaded area indicates concentrations below dolutegravir in-vivo EC_90_. The dashed lines indicate published geometric mean adult reference values for 50 mg onc per day (lower lines) and twice per day (upper lines). DT=dispersible tablet. FCT=film-coated tablet. AUC_0–24 h_=area under the concentration-time curve from 0 to 24 h. C_trough_=trough concentration. C_max_=maximum concentration. EC_90_=the effective concentration at which 90% of maximal viral inhibition is achieved in a 10-day monotherapy study. *Children younger than 6 months.

The geometric mean C_trough_ in the 3 kg to less than 6 kg weight band and younger than 6 months was 0·64 mg/L (coefficient of variation 51%), 6 kg to less than 10 kg was 0·53 mg/L (150%), and 10 kg to less than 14 kg was 0·77 mg/L (57%). In children weighing 14 kg to less than 20 kg taking 25 mg of film-coated tablets, the geometric mean C_trough_ was 0·44 mg/L (coefficient of variation 50%) whereas those taking 25 mg of dispersible tablets had a higher geometric mean C_trough_ of 0·87 mg/L (64%). Compared with adults taking 50 mg dolutegravir once per day, higher variability was seen in all weight bands, but was highest in the 6 kg to less than 10 kg weight band (table 2). Overall, eight children had a C_trough_ below the EC_90_ of 0·32 mg/L: four (31%) of 13 in the 6 kg to less than 10 kg weight band receiving 15 mg dispersible tablets and four (21%) of 19 in the 14 kg to less than 20 kg weight band receiving 25 mg film-coated tablets (figure 3). All C_trough_ values remained above the minimum value for in-vitro inhibition IC_90_ (ie, 0·064 mg/L).

For all weight bands, the geometric mean AUC_0–24 h_ was comparable to or higher than reference values of adults on the 50 mg film-coated tablets once per day and lower than reference values of adults on the 50 mg film-coated tablets twice per day. A higher variability in children than in adults taking the 50 mg once per day dose was reported (table 2). In children younger than 6 months and in the 3 kg to less than 6 kg weight band and in those within the 14 kg to less than 20 kg weight band taking film-coated tablets, the geometric mean C_max_ was between reference values of adults taking the 50 mg film-coated tablets once per day and twice per day. The geometric mean C_max_ was 3·7% higher in the 6 kg to less than 10 kg weight band, 49·0% higher in the 10 kg to less than 14 kg weight band, and 33·1% higher in the 14 kg to less than 20 kg weight band taking dispersible tablets than the geometric mean C_max_ in adults taking 50 mg film-coated tablets twice per day (table 2).

Nine children in the 14 kg to less than 20 kg weight band had a pharmacokinetic curve on both dolutegravir 25 mg film-coated tablet and 25 mg dispersible tablet. A mixed effects model, including all profiles of the 14 kg to less than 20 kg weight band with film-coated tablets as the reference group and dispersible tablets as the comparator group, resulted in a geometric mean ratio for C_trough_ of 1·94 (90% CI 1·40–2·71), AUC_0–24 h_ of 1·73 (1·40–2·14), oral clearance of 0·58 (0·47–0·71), and C_max_ of 1·78 (1·48–2·13).

Virological outcomes were examined for the 36 children who started and completed pharmacokinetic profiles on a dispersible dolutegravir formulation (n=5 for 5mg, n=1 for 10 mg, n=13 for 15 mg, n= 11 for 20mg, and n=7 for 25 mg), including one child who completed pharmacokinetic profiles on two doses (15 mg while weighing 6 kg to <10 kg [C_trough_ of 0·15 mg/L] and 20 mg while weighing 10 kg to <14 kg [C_trough_ of 0·67 mg/L]). Of the 36 children, 28 (78%) had viral loads less than 400 copies per mL at week 48, of whom four had a C_trough_ below the EC_90_. All four children were in the 6 kg to less than 10 kg weight band receiving 15 mg dispersible tablets. Eight (22%) of 36 children had viral loads of 400 copies per mL or more at week 48, including five who had confirmed viral loads of 400 copies per mL or more (two consecutive viral loads of ≥400 copies per mL); all eight children had a C_trough_ above EC_90_.

Safety data are presented in 71 children consenting to participate in the pharmacokinetic substudies after excluding one for major ineligibility. 13 children in the 14 kg to less than 20 kg weight band participated in substudy one (25 mg film-coated tablets) and substudy two (25 mg dispersible tablets); all children completed 24 weeks follow-up before entering substudy two. Two children completed a pharmacokinetic profile on a non-protocol dose and were switched to a correct per-protocol dose; a repeat pharmacokinetic profiling was done and safety data to 24 weeks from starting the correct dose were collected. One child completed pharmacokinetic profiling on two per-protocol doses with total safety follow-up of 36 weeks (24 weeks from starting the second dose). One child withdrew at 5 weeks during safety follow-up. 19 (27%) of 71 children had 29 reportable grade 3 or higher adverse events. Overall, 11 (15%) of 71 children had 13 serious adverse events, including two deaths (table 3; appendix pp 6–9). Primarily, the grade 3 or higher adverse events were due to infectious diseases (15 [52%] of 29 events) or asymptomatic laboratory abnormalities (eight [28%]). One child discontinued dolutegravir because of raised liver enzyme concentrations, later attributable to hepatitis A virus infection. No other events resulted in modification of ART.

**Table 3:** Summary of adverse events by weight band and dolutegravir dose and formulation

		**Total**	**3 kg to <6 kg (<6 months)**	**3 kg to <6 kg (≥6 months)**	**6 kg to <10 kg**	**10 kg to <14 kg**	**14 kg to <20 kg**	**14 kg to <20 kg**	**20 kg to <25 kg**	**Non-per protocol**
Dose*		..	5 mg	10 mg	15 mg	20 mg	25 mg	25 mg	30 mg	..
Formulation		..	DT	DT	DT	DT	FCT	DT	DT	..
Number of participants		71	8	5	28	18	25	19	2	14
Follow-up, weeks†										
	Median	24·0 (24·0–24·0)	11·3 (6·6–12·0)	10·9 (4·0–12·0)	15·6 (12·0–24·0)	24·0 (12·0–24·0)	24·0 (24·0–24·0)	24·0 (23·6–24·0)	11·3 (0·4–22·1)	5·4 (1·9–11·7)
	Range	1·0–48·0	1·3–12·0	1·0–20·1	0·0–24·0	0·0–24·0	12·7–24·0	0·6–24·0	0·4–22·1	0·1–23·4
Number of participants with a serious adverse event		11/71 (15%)	2/8 (25%)	2/5 (40%)	3/28 (11%)	1/18 (6%)	2/25 (8%)	0	0	1/14 (7%)
Number of serious adverse events‡		13	2	2	3	1	2	0	0	3
	Haematological	1 (8%)	1 (50%)	0	0	0	0	0	0	0
	Infectious disease	9 (69%)	1 (50%)	1 (50%)	2 (67%)	1 (100%)	2 (100%)	0	0	2 (67%)
	Non-HIV-related deaths (traumatic)	1 (8%)	0	0	0	0	0	0	0	1 (33%)§
	Systemic	2 (15%)	0	1 (50%)¶	1 (33%)	0	0	0	0	0
Number of participants with a grade ≥3 event		19/71 (27%)	2/8 (25%)	2/5 (40%)	5/28 (18%)	4/18 (22%)	3/25 (12%)	1/19 (5%)	0	2/14 (14%)
Number of grade ≥3 events‖		29	3	3	7	8	3	1	0	4
	Biochemical	2 (7%)	0	0	0	2 (25%)	0	0	0	0
	Haematological	7 (24%)	2 (67%)	0	1 (14%)	3 (38%)	0	0	0	1 (25%)
	Infectious disease	15 (52%)	1 (33%)	1 (33%)	5 (71%)	3 (38%)	2 (67%)	1 (100%)**	0	2 (50%)
	Nervous system	1 (3%)	0	0	0	0	1 (33%)	0	0	0
	Non-HIV-related deaths (traumatic)	1 (3%)	0	0	0	0	0	0	0	1 (25%)§
	Systemic	3 (10%)	0	2 (67%)¶	1 (14%)	0	0	0	0	0
Person-years		38·4	1·4	0·9	8·9	6·3	11·2	7·2	0·4	2·1
Grade ≥3 event rate per 100 person-years (95% CI)		76 (51–108)	214 (44–625)	326 (67–953)	79 (32–163)	127 (55–251)	27 (6–79)	14 (0–77)	0 (0–0)	194 (53–495)

Data are n, median (IQR), n/N (%), or n (%), unless otherwise specified. 13 children participated in the first (25 mg film-coated tablet) and second (25 mg dispersible tablet) pharmacokinetic substudies (14 kg to <20 kg) with no overlap; one child was exposed to and completed pharmacokinetic profiles on dispersible 15 mg while weighing 6 kg to less than <10 kg (12 weeks safety follow-up; two grade ≥3 events reported as components of the same clinical serious adverse event) and 20 mg while weighing 10 kg to less than <14 kg (24 weeks safety follow-up; no events reported); one child was exposed to and completed pharmacokinetic profiles on dispersible 10 mg (non-per-protocol dose; 3 weeks safety follow-up) and 5 mg (24 weeks safety follow-up) while weighing 3 kg to less than 6 kg and being younger than 6 months (no events reported); one child was exposed to and completed pharmacokinetic profiles on 20 mg film-coated tables (non-per-protocol dose; 21 weeks safety follow-up) and 25 mg dispersible tablet (24 weeks safety follow-up) while weighing 14 kg to less than 20 kg (no events reported). DT=dispersible tablet. FCT=film-coated tablet.

* Exposed to dolutegravir once per day or twice per day with rifampicin: one child weighing 3 kg to less than 6 kg and being younger than 6 months was exposed to 10 mg dispersible dolutegravir twice per day (non-per-protocol dose); three children weighing 6 kg to less than 10 kg were exposed to 15 mg dispersible dolutegravir twice per day; two children weighing 10 kg to less than 14 kg were exposed to 20 mg dispersible dolutegravir twice per day; one child weighing 14 kg to less than 20 kg was exposed to 25 mg dispersible dolutegravir twice per day; and two children weighing 14 kg to less than 20 kg were exposed to 25 mg film-coated dolutegravir twice per day.

† Follow-up based on time-updated dose: dose changes were primarily the result of increased weight and moving to the intended pharmacokinetic dose in the second pharmacokinetic substudy.

‡ Serious adverse events are analysed as episodes, with all components of the same clinical serious adverse events presented as one episode.

§ One child died due to a traumatic accident.

¶ One child died due to kwashiorkor.

‖ For grade 3 or higher clinical and laboratory adverse events, each component of the same episode is analysed as a separate event.

** One event (ie, hepatitis A virus infection) resulted in discontinuation of dolutegravir.

Two children in the 3 kg to less than 6 kg weight band died; the cause of death was kwashiorkor at 1 week after randomisation in a child older than 6 months receiving 10 mg dispersible tablets and a traumatic accident at 18 weeks in a child younger than 6 months and receiving a non-per-protocol dose of 10 mg dispersible tablets twice per day with rifampicin (table 3). The reporting clinicians and endpoint review committee considered none of the grade 3 or higher adverse events to be related to dolutegravir exposure.

Over a total follow-up of 25·2 person-years in 59 children exposed to dispersible tablets (on the per-protocol doses), 22 grade 3 or higher adverse events were reported (87·4 per 100 person-years, 95% CI 54·8–132·4); although these children were mostly less than 14 kg where event rates are numerically higher than in older children weighing more than 14 kg (table 3). Over a total follow-up of 11·2 person-years in 25 children weighing more than 14 kg exposed to film-coated tablets (on per-protocol doses), three grade 3 or higher adverse events were reported (26·9 per 100 person-years, 95% CI 5·5–78·6). No significant evidence of an association between log AUC_0–24h_ or log C_max_ and occurrence of adverse events in children were reported (appendix pp 10–11).

## Discussion

Data from these pharmacokinetic substudies nested within the ODYSSEY trial supported licensing of dolutegravir 5 mg dispersible formulations in children older than 4 weeks and weighing 3 kg to less than 20 kg. The study showed acceptable pharmacokinetic parameters and safety using increasing number of dispersible tablets across WHO weight bands in children weighing 3 kg to less than 20 kg. A dolutegravir dose of 25 mg film-coated tablet for children weighing 14 kg to less than 20 kg was insufficient to reach pharmacokinetic targets comparable to adults.

In this study, we used C_trough_ as the primary pharmacokinetic target based on data from in-vitro and in-vivo studies suggesting a correlation between C_trough_ and virological effect.^12^, ^17^ The use of C_trough_ as the primary pharmacokinetic target for viral load reduction was reinforced by virological outcomes at 48 weeks of treatment in a treatment-experienced population.^19^ We compared geometric mean C_trough_ in each weight band to the adult reference value of 0·83 mg/L and considered the percentage of children with C_trough_ below EC_90_ for each weight band.^12^ This adult target has been extrapolated for children, considering the similar course of HIV infection and the effects of antiretroviral drugs in adults and children.^20^, ^21^ Stringent regulatory authorities also consider geometric mean C_trough_ results comparable to adults as a key parameter in determining an adequate exposure to dolutegravir in children.

Our results show that C_trough_ was comparable to the adult reference for all weight bands of children weighing 3 kg to less than 20 kg taking dolutegravir dispersible tablets with the exception of those in the 6 kg to less than 10 kg weight band. In this weight band the geometric mean C_trough_ was 36% lower than the adult reference value and 31% of children in this group did not achieve C_trough_ above EC_90_, although all C_trough_ did remain above IC_90_, suggesting that the virus should theoretically still be suppressed. Additionally, we found no evidence that children with C_trough_ below EC_90_ were less likely to be suppressed at week 48 than those with higher C_trough_. IMPAACT P1093, a dolutegravir paediatric dose-finding study, explored the same doses in weight bands as ODYSSEY and reported geometric mean C_trough_ in children weighing 6 kg to less than 10 kg on 15 mg dispersible tablets dolutegravir comparable to adult reference values.^22^ A population-pharmacokinetic model based on both P1093 and ODYSSEY data confirmed that the 15 mg dispersible tablet dose provided optimal exposure for this weight band.^23^ Both studies had high variability in this weight band, possibly because of age-related enzyme maturation that might not be fully developed in some participants and variability in nutritional status.^15^, ^24^

In children weighing 14 kg to less than 20 kg taking film-coated tablets, we observed a 47% lower geometric mean C_trough_ compared with the adult reference value, and 21% of children in this group did not achieve a C_trough_ above EC_90_. The results inferred that the dose of 20 mg film-coated tablets previously licensed by EMA for this weight band was suboptimal in our population. Based on the results of this study, the 20 mg film-coated tablets are no longer recommended by regulatory authorities for children weighing 14 kg to less than 20 kg.

Our study design gave us an opportunity to investigate the relative bioavailability in a mixed intrapatient and interpatient comparison in children taking 25 mg film-coated tablets and 25 mg dispersible tablets. On 25 mg dispersible tablets, children achieved adequate geometric mean C_trough_. Geometric mean AUC_0–24 h_ increased by a ratio of 1·73, indicating higher bioavailability of the dispersible tablets. This ratio is comparable to that seen in adult bioequivalence studies with dispersible versus film-coated tablets.^15^ The geometric mean ratio reported in adults taking 25 mg dispersible tablets versus 25 mg film-coated tablets was 1·56 for C_trough_, 1·62 for AUC_0–24 h_, and 1·79 for C_max_ (appendix p 12).^15^, ^18^ Using a relative bioavailability of 1·6, the equivalent film-coated tablet dose to 25 mg dispersible tablets is 40 mg, which is now the recommended dose in absence of dispersible tablets in the 14 kg to less than 20 kg weight band.

Pharmacokinetic evaluations in this study were done in a fasted state to enable comparison with the adult fasted reference values. For children weighing less than 10 kg, fasting 2 h before the dolutegravir dose and 1 h after the dose was preferred, but not mandatory. In our study, only one child weighing less than 10 kg was unable to adhere to the preferred fasting. Taking dolutegravir with a meal has been shown to increase C_trough_ up to 73% in adults; thus, the pharmacokinetic results presented here possibly simulate the scenario with the lowest drug concentrations.^25^

Our study showed reassuring safety data for dolutegravir in children weighing 3 kg to less than 20 kg. Although relatively more adverse events were reported in children weighing less than 14 kg, the numbers are small and most adverse events were the result of infectious illnesses that are very common in infants in low-income and middle-income countries and are not unexpected. None of the adverse events were related to dolutegravir as reported by the site investigators or adjudicated by the Endpoint Review Committee masked to the treatment allocation. Although peak concentrations were higher than the adult reference in three weight bands taking dispersible tablets, no significant association with increased adverse events was observed for increased geometric mean C_max_ or geometric mean AUC_0–24 h_ concentrations. Overall, dolutegravir was well tolerated and we identified no new safety issues in children.

Apart from during the pharmacokinetic assessment days, children were allowed to take their antiretroviral drugs with and without food; and given the effect of food on dolutegravir concentrations, the safety data presented here reflect the real-life situation. Dispersible tablets are the preferred type of drug formulation for infants and young children who are unable to swallow tablets. This formulation is easily stored and transported and can be scaled to accommodate an increase in dosing in a growing child. The data from this study have contributed to the recent decisions to license 5 mg dolutegravir dispersible tablets for use in children from 4 weeks of age weighing 3 kg to less than 20 kg by the US Food and Drug Administration (FDA) and EMA, and informed the current WHO antiretroviral guidelines.^3^, ^26^, ^27^ Through a collaboration between Unitaid, Clinton Health Access Initiative, and ViiV Healthcare, generic dolutegravir dispersible tablets, bioequivalent to the dispersible tablets used in this study, are becoming available for low-income and middle-income countries at low cost, expecting to cut expenses for HIV treatment in these countries by 75%.^28^

A limitation of our study was that the desired sample size was not reached for children weighing 3 kg to less than 6 kg younger than 6 months (five evaluable, eight targeted) and for children weighing 3 kg to less than 6 kg older than 6 months (one evaluable, eight targeted). Higher than expected coefficient of variation percentages in all weight bands could also have justified increasing our target sample sizes. However, pharmacokinetic and safety studies were also ongoing in IMPAACT and the population pharmacokinetic modelling of combined IMPAACT and ODYSSEY data from 239 children across all weight bands and ages was sufficient to support regulatory approval of dispersible tablet dosing in weight bands from 3 kg to less than 20 kg including a 5 mg dispersible tablet in the 3 kg to less than 6 kg weight band, irrespective of age.^15^, ^23^ In ODYSSEY, we had four children who were prescribed an incorrect dose in this weight band, suggesting that a differential dose by age is impractical and prone to prescribing errors. This finding supports the FDA approval of a single dose for children in this weight band.

The results presented in this paper together with the results of the ODYSSEY pharmacokinetic substudies in children weighing 20 kg or more showed that dolutegravir dosing in children across all weight bands can be achieved with two formulations: paediatric 5 mg (or scored 10 mg) dispersible tablets and adult 50 mg film-coated tablets.^5^ Access to a minimal number of effective formulations and adult formulation down to the lowest possible weight band supported by pharmacokinetic and safety data will improve availability of antiretroviral drugs and reduce fragmentation of the paediatric antiretroviral drug market. Harmonisation of adult and paediatric antiretroviral treatment will simplify drug procurement and prescribing, and has become a key goal for treatment optimisation.^29^, ^30^

In conclusion, WHO weight band-based dosing with dolutegravir dispersible tablets achieves adequate dolutegravir exposure in most children weighing 3 kg to less than 20kg with reassuring safety profiles. The data in this study have contributed to the approval of dolutegravir 5 mg dispersible tablets by stringent regulatory authorities and informed recently updated WHO paediatric antiretroviral dosing and formulation guidelines.^3^, ^26^, ^27^, ^29^ Moreover, once dolutegravir dispersible formulations produced by generic companies are available in low-income and middle-income countries, global access to dolutegravir for children older than 4 weeks will be possible.

## Data sharing

The ODYSSEY data are held at the Medical Research Council Clinical Trials Unit at University College London, which encourages optimal use of data by using a controlled access approach to data sharing, incorporating a transparent and robust system to review requests and provide secure data access consistent with the relevant ethics committee approvals. All requests for data are considered and can be initiated by contacting mrcctu.ctuenquiries@ucl.ac.uk.

## Declaration of interests

DMB has received payments for serving on an advisory board of ViiV Healthcare. DMB, PDJB, HW, and AC have received research funding for Radboudumc Institute for Health Sciences from ViiV Healthcare. All other authors declare no competing interests.
