# Supplementary material for: Dolutegravir dosing for children with HIV weighing less than 20 kg: pharmacokinetic and safety substudies nested in the open-label, multicentre, randomised, non-inferiority ODYSSEY trial
Source: Lancet HIV. 2022 Feb 18;9(5):e341–52. doi: 10.1016/S2352-3018(21)00292-7 (PMC9046096; doi:10.1016/S2352-3018(21)00292-7)
Supplement: Supplementary appendix [file mmc1.pdf]

THE LANCET
HIV

Supplementary appendix

This appendix formed part of the original submission and has been peer reviewed. We post it as supplied by the authors.

Supplement to: Waalewijn H, Chan M K, Bollen P D J, et al. Dolutegravir dosing for children with HIV weighing less than 20 kg: pharmacokinetic and safety substudies nested in the open-label, multicentre, randomised, non-inferiority ODYSSEY trial. *Lancet HIV* 2022; published online Feb 18. https://doi.org/10.1016/S2352-3018(21)00292-7.

Dolutegravir dosing for children with HIV weighing less than 20 kg: pharmacokinetic and safety substudies nested in the multicentre, randomised ODYSSEY trial.

Supplementary appendix

Content

1. Supplementary table 1. Dolutegravir dosing and administration details in the pharmacokinetic substudies for children weighing 3 to less than 20kg
2. Supplementary table 2. Children in Weight band 3 to <6kg older than 6 months taking 10mg dispersible tablet, not reported in main paper due to limited data of this group
3. Supplementary figure 1. Individual dolutegravir C_{trough}, $AUC_{0–24h}$, and C_{max} in children weighing 3 kg to less than 20 kg taking dispersible tablets or film-coated tablets, including one child weighing 3 to less than 6kg taking 10mg dispersible tablets
4. Supplementary figure 2. Surface plot showing dolutegravir plasma concentrations by participant weight and time since dolutegravir dosing for all participants receiving dolutegravir dispersible tablets
5. Supplementary table 3. Individual PK parameters of children ineligible for pharmacokinetic analysis with reasons for exclusion
6. Supplementary table 4. Detailed summary of adverse events by weight band and dolutegravir dose and formulation
7. Supplementary table 5. Listing of adverse events reported
8. Supplementary table 6. Association between log AUC_{0-24h} or log C_{max} and occurrence of adverse events
9. Supplementary figure 3. Log $AUC_{\mathbf{0-24h}}$ in children with vs without adverse events reported overall and by weight band
10. Supplementary figure 4. Log C_{max} in children with vs without adverse events reported overall and by weight band
11. Supplementary figure 5. Supplementary figure 5. Intra- and inter-subject comparison of pharmacokinetic parameters on 25mg film-coated tablets (FCT; reference) and 25mg dispersible tablets (DT; test)
12. The Odyssey trial team

Supplementary Table 1. Dolutegravir dosing and administration details in the pharmacokinetic substudies for children weighing 3 to less than 20kg

Weight band	Dose in mg	Formulation type and amount of tablets	Administration details	Food restrictions	Blood draw volume
3 to <6kg; <six months old	5	1 dispersible tablet	Dispersed in 10 mL water; rinsed with 10 mL water	Preferred fasting: 2 hours before dose, 1 hour after dose	0·5 ml sample at 7 time points
3 to <6kg; >six months old	10	2 dispersible tablets	Dispersed in 10 mL water; rinsed with 10 mL water	Preferred fasting: 2 hours before dose, 1 hour after dose	0·5 ml sample t 7 time points
6 to <10kg	15	3 dispersible tablets	Dispersed in 10 mL water; rinsed with 10 mL water	Preferred fasting: 2 hours before dose, 1 hour after dose	1 ml sample at 7 time points
10 to <14kg	20	4 dispersible tablets	Dispersed in 10 mL water; rinsed with 10 mL water	Mandatory fasting: At least 3 hours before dose; 2 hours after dose	2 ml sample at 7 time points
14 to <20kg	25	5 dispersible tablets	Dispersed in 15 mL water; rinsed with 10 mL water, followed up with 75 mL water (100 mL in total)	Mandatory fasting: at least 3 hours before dose; 2 hours after dose	2 ml sample at 7 time points
14 to <20kg	25	1 film-coated tablet	Taken with 100 mL water	Mandatory fasting: at least 3 hours before dose; 2 hours after dose	2 ml sample at 7 time points

Supplementary table 2. Children in Weight band 3 to <6kg older than 6 months taking 10mg dispersible tablet, not reported in main paper due to limited data of this group.

ID	WEIGHT (KG)	AGE (YEARS)	DOSE (MG)	FORMULATION	CTROUGH (MG/L)	AUC0-24 (H*MG/L)	CMAX (MG/L)	CL/F (L/H)	T1/2 (H)	VD/F (L)
**1**	5·7	0·61	10	DT	1·77	103·20	8·00	0·10	10·36	1·45

C_{trough}: dolutegravir trough concentration, AUC_{0-24}: area under the concentration-time curve from 0 to 24 hours. C_{max}: maximum concentration, CL/F: apparent clearance. DT: dispersible tablet

Supplementary figure 1. Individual dolutegravir Ctrough, AUC0–24h, and Cmax in children weighing 3 kg to less than 20 kg taking dispersible tablets (DT) or film-coated tablets (FCT), including one child weighing 3 to less than 6kg older than 6 months taking 10mg dispersible tablets.

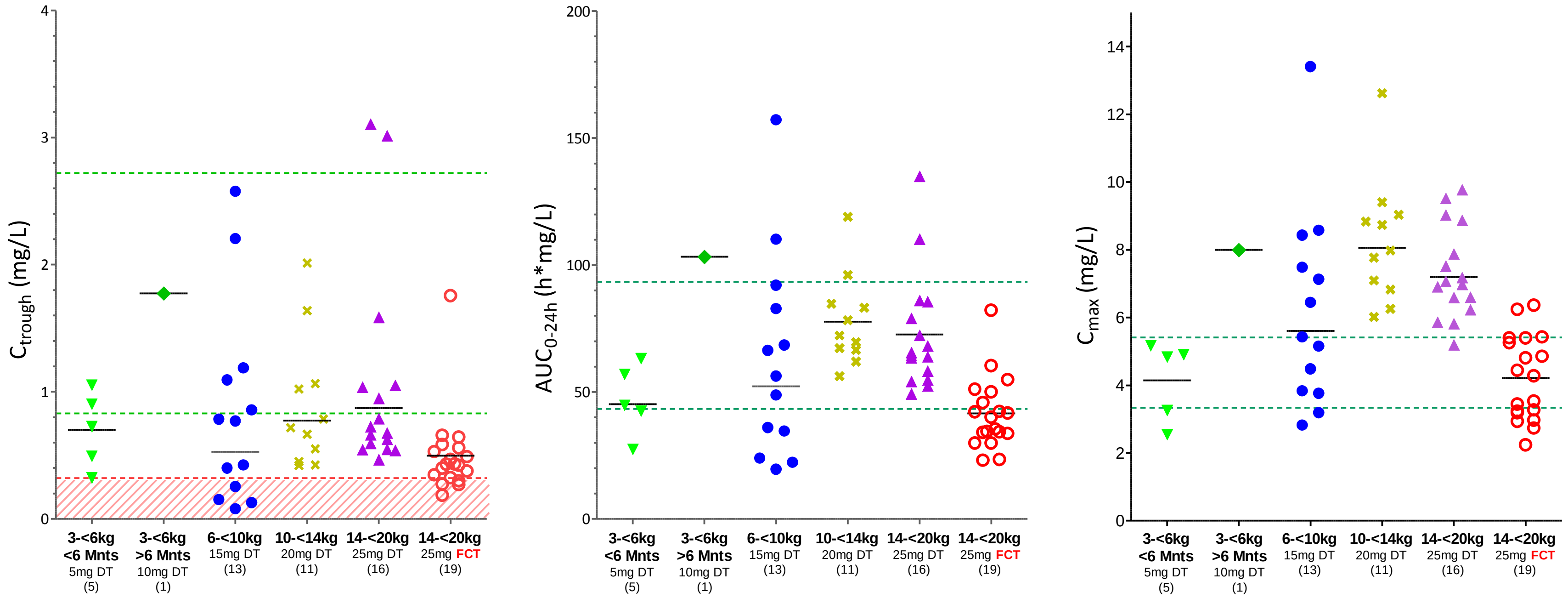

Horizontal black lines indicate geometric means per dose. Red line indicates dolutegravir in-vivo EC90. Green dashed lines indicate published geometric mean adult reference values for 50 mg once-daily (lower line) and twice-daily doses (upper line). Ctrough=trough concentration. AUC0–24h=area under the concentration-time curve from 0 to 24 h. Cmax=maximum concentration. EC90=the effective concentration at which 90% of maximal viral inhibition is achieved in a 10-day monotherapy study.

Supplementary figure 2. Surface plot showing dolutegravir plasma concentrations by participant weight and time since dolutegravir dosing for all participants receiving dolutegravir dispersible tablets.

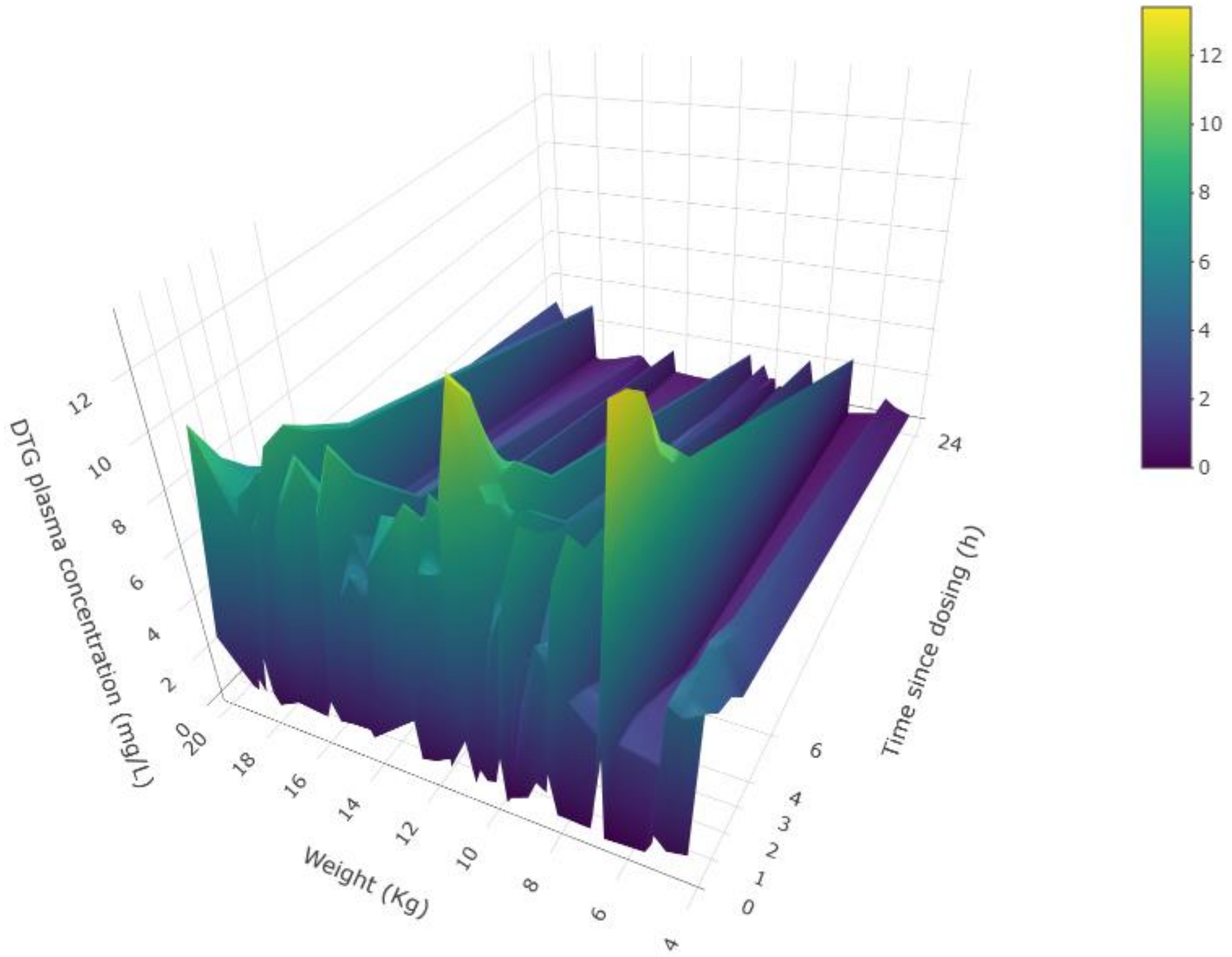

Time since dolutegravir dosing (0, 1, 2, 3, 4, 6 and 24 hours)

Supplementary table 3. Individual PK parameters of children ineligible for pharmacokinetic analysis with reasons for exclusion

ID	WEIGHT (KG)	AGE (YEARS)	DOSE (MG)	FORMULATION	C_{TROUGH} (MG/L)	AUC_{0-24} (H*MG/L)	C_{MAX} (MG/L)	CL/F (L/H)	$T_{1/2}$ (H)	V_D/F (L)	REASON FOR EXCLUSION
**1**	4·60	0·29	10	DT	1·41	88·95	7·60	0·11	9·11	1·48	Wrong dose for age and weight band
**2**	4·90	0·52	10	DT	0·49	32·72	2·62	0·31	8·96	3·95	Concomitant medication (Zinc Sulphate)
**3**	5·20	0·61	10	DT	1·10	80·57	6·92	0·12	8·17	1·46	Non-adherence
**4**	5·30	0·43	10	DT	4·62	180·64	12·29	0·06	17·65	1·41	Wrong dose for age and weight band
**5**	7·00	0·42	10	DT	2·43	127·60	9·47	0·08	11·17	1·26	Wrong dose for weight band
**6**	7·80	2·65	15	DT	1·07	78·27	7·46	0·19	7·91	2·19	Non-adherence
**7**	8·70	1·98	15	DT	0·54	54·15	5·24	0·28	6·47	2·59	Non-adherence
**8**	10·20	1·47	20	DT	1·06	73·21	6·55	0·27	8·66	3·41	Participant did not fast
**9**	11·90	2·66	20	DT	0·80	61·76	5·84	0·32	7·90	3·69	Non-adherence
**10**	14·70	4·68	25	FCT	0·95	56·14	4·33	0·45	9·99	6·42	DTG intake out of allowed dosing window
**11**	15·00	5·13	25	FCT	c.n.b.d.	c.n.b.d.	6.09	c.n.b.d.	c.n.b.d.	c.n.b.d.	DTG intake out of allowed dosing window
**12**	16·40	5·58	20	FCT	0·56	38·55	3·56	0·52	8·42	6·30	Wrong dose for weight band
**13**	17·00	7·61	25	FCT	0·06	8·95	1·48	2·79	5·75	23·18	Concomitant medication (iron supplement)
**14**	18·30	4·37	20	FCT	0·20	21·46	2·43	0·93	6·45	8·67	Major ineligibility as child was enrolled as ART-naïve when was taking ART supplied by an alternative clinic
**14**	19·00	4·96	25	DT	0·58	51·50	5·56	0·49	7·19	5·04	Major ineligibility as child was enrolled as ART-naïve when was taking ART supplied by an alternative clinic
**15**	18·50	8·85	25	FCT	0·09	21·32	3·63	1·17	4·67	7·90	Non-adherence
**16**	18·80	5·38	25	DT	0·18	35·11	4·52	0·71	4·85	4·98	Concomitant medication (valproic acid)
**17**	20·10	7·48	25	DT	1·47	84·70	6·88	0·30	9·65	4·11	DTG intake out of allowed dosing window

C_{trough}: dolutegravir trough concentration, AUC_{0-24}: area under the concentration-time curve from 0 to 24 hours. C_{max}: maximum concentration, CL/F: apparent clearance, C.n.b.d.: could not be determined· DT: dispersible tablet, FCT: film-coated tablet, DTG: dolutegravir. Dosing window: between 20 and 28 hours after previous DTG dose.

Supplementary table 4. Detailed summary of adverse events by weight band and dolutegravir dose and formulation

	Total	3-<6kg,<6m	3-<6kg,≥6m	6-<10kg	10-<14kg	14-<20kg		20-<25kg	Non-Per Protocol
**Dose‡**	-	5mgDT	10mgDT	15mgDT	20mgDT	25mgFCT	25mgDT	30mgDT	-
**Follow-up$^{\alpha}$, weeks**									
**No. of Participants**	**71**	**8**	**5**	**28**	**18**	**25**	**19**	**2**	**14**
Median	24·0	11·3	10·9	15·6	24·0	24·0	24·0	11·3	5·4
(IQR)	(24·0-24·0)	(6·6-12·0)	(4·0-12·0)	(12·0-24·0)	(12·0-24·0)	(24·0-24·0)	(23·6-24·0)	(0·4-22·1)	(1·9-11·7)
[Range]	[1·0-48·0]	[1·3-12·0]	[1·0-20·1]	[0·0-24·0]	[0·0-24·0]	[12·7-24·0]	[0·6-24·0]	[0·4-22·1]	[0·1-23·4]
**Number of SAEs (No. of Participants)##**	**13 (11)**	**2 (2)**	**2 (2)**	**3 (3)**	**1 (1)**	**2 (2)**	**0 (0)**	**0 (0)**	**3 (1)**
**Haematological**	**1 (1)**	**1 (1)**	**0 (0)**	**0 (0)**	**0 (0)**	**0 (0)**	**0 (0)**	**0 (0)**	**0 (0)**
Thrombocytopenia+Neutropenia	1 (1)	1 (1)	0 (0)	0 (0)	0 (0)	0 (0)	0 (0)	0 (0)	0 (0)
**Infectious Disease**	**9 (8)**	**1 (1)**	**1 (1)**	**2 (2)**	**1 (1)**	**2 (2)**	**0 (0)**	**0 (0)**	**2 (1)**
Acute diarrhoea not investigated+Dehydration	1 (1)	0 (0)	1 (1)	0 (0)	0 (0)	0 (0)	0 (0)	0 (0)	0 (0)
Acute febrile episode - undiagnosed	1 (1)	0 (0)	0 (0)	0 (0)	0 (0)	1 (1)	0 (0)	0 (0)	0 (0)
Chronic diarrhoea with no pathogen	1 (1)	0 (0)	0 (0)	1 (1)	0 (0)	0 (0)	0 (0)	0 (0)	0 (0)
Gastroenteritis	1 (1)	1 (1)	0 (0)	0 (0)	0 (0)	0 (0)	0 (0)	0 (0)	0 (0)
Measles+Chest infection	1 (1)	0 (0)	0 (0)	0 (0)	1 (1)	0 (0)	0 (0)	0 (0)	0 (0)
Plasmodium falciparum malaria	1 (1)	0 (0)	0 (0)	0 (0)	0 (0)	1 (1)	0 (0)	0 (0)	0 (0)
Pneumonia no organism identified	1 (1)	0 (0)	0 (0)	0 (0)	0 (0)	0 (0)	0 (0)	0 (0)	1 (1)
Pneumonia no organism identified+acute otitis media	1 (1)	0 (0)	0 (0)	1 (1)	0 (0)	0 (0)	0 (0)	0 (0)	0 (0)
Tuberculosis - pulmonary - smear negative or not done	1 (1)	0 (0)	0 (0)	0 (0)	0 (0)	0 (0)	0 (0)	0 (0)	1 (1)
**Non HIV related deaths**	**1 (1)**	**0 (0)**	**0 (0)**	**0 (0)**	**0 (0)**	**0 (0)**	**0 (0)**	**0 (0)**	**1 (1)**
Traumatic *	1 (1)	0 (0)	0 (0)	0 (0)	0 (0)	0 (0)	0 (0)	0 (0)	1 (1)
**Systemic**	**2 (2)**	**0 (0)**	**1 (1)**	**1 (1)**	**0 (0)**	**0 (0)**	**0 (0)**	**0 (0)**	**0 (0)**
Chest infection+Severe malnutrition	1 (1)	0 (0)	0 (0)	1 (1)	0 (0)	0 (0)	0 (0)	0 (0)	0 (0)
Kwashiorkor *	1 (1)	0 (0)	1 (1)	0 (0)	0 (0)	0 (0)	0 (0)	0 (0)	0 (0)
**Number of grade ≥3 events (No. of Participants)###**	**29 (19)**	**3 (2)**	**3 (2)**	**7 (5)**	**8 (4)**	**3 (3)**	**1 (1)**	**0 (0)**	**4 (2)**
**Biochemical**	**2 (2)**	**0 (0)**	**0 (0)**	**0 (0)**	**2 (2)**	**0 (0)**	**0 (0)**	**0 (0)**	**0 (0)**
Raised AST	1 (1)	0 (0)	0 (0)	0 (0)	1 (1)	0 (0)	0 (0)	0 (0)	0 (0)
Raised alkaline phosphatase (ALK)	1 (1)	0 (0)	0 (0)	0 (0)	1 (1)	0 (0)	0 (0)	0 (0)	0 (0)
**Haematological**	**7 (5)**	**2 (1)**	**0 (0)**	**1 (1)**	**3 (2)**	**0 (0)**	**0 (0)**	**0 (0)**	**1 (1)**
Anaemia with no clinical symptoms	4 (4)	0 (0)	0 (0)	1 (1)	2 (2)	0 (0)	0 (0)	0 (0)	1 (1)
Neutropenia	2 (2)	1 (1)	0 (0)	0 (0)	1 (1)	0 (0)	0 (0)	0 (0)	0 (0)
Thrombocytopenia	1 (1)	1 (1)	0 (0)	0 (0)	0 (0)	0 (0)	0 (0)	0 (0)	0 (0)

**Infectious Disease**	**15 (12)**	**1 (1)**	**1 (1)**	**5 (4)**	**3 (2)**	**2 (2)**	**1 (1)**	**0 (0)**	**2 (1)**
Acute diarrhoea not investigated	2 (2)	0 (0)	1 (1)	1 (1)	0 (0)	0 (0)	0 (0)	0 (0)	0 (0)
Acute febrile episode undiagnosed	1 (1)	0 (0)	0 (0)	0 (0)	0 (0)	1 (1)	0 (0)	0 (0)	0 (0)
Acute otitis media	1 (1)	0 (0)	0 (0)	1 (1)	0 (0)	0 (0)	0 (0)	0 (0)	0 (0)
Chest infection	2 (2)	0 (0)	0 (0)	1 (1)	1 (1)	0 (0)	0 (0)	0 (0)	0 (0)
Chronic diarrhoea with no pathogen	1 (1)	0 (0)	0 (0)	1 (1)	0 (0)	0 (0)	0 (0)	0 (0)	0 (0)
Gastroenteritis	1 (1)	1 (1)	0 (0)	0 (0)	0 (0)	0 (0)	0 (0)	0 (0)	0 (0)
Hepatitis A	2 (2)	0 (0)	0 (0)	0 (0)	1 (1)	0 (0)	1 (1)**	0 (0)	0 (0)
Measles	1 (1)	0 (0)	0 (0)	0 (0)	1 (1)	0 (0)	0 (0)	0 (0)	0 (0)
Plasmodium falciparum malaria	1 (1)	0 (0)	0 (0)	0 (0)	0 (0)	1 (1)	0 (0)	0 (0)	0 (0)
Pneumonia no organism identified	2 (2)	0 (0)	0 (0)	1 (1)	0 (0)	0 (0)	0 (0)	0 (0)	1 (1)
Tuberculosis - pulmonary - smear negative or not done	1 (1)	0 (0)	0 (0)	0 (0)	0 (0)	0 (0)	0 (0)	0 (0)	1 (1)
**Nervous System**	**1 (1)**	**0 (0)**	**0 (0)**	**0 (0)**	**0 (0)**	**1 (1)**	**0 (0)**	**0 (0)**	**0 (0)**
Epilepsy, fits, convulsions	1 (1)	0 (0)	0 (0)	0 (0)	0 (0)	1 (1)	0 (0)	0 (0)	0 (0)
**Non HIV related deaths**	**1 (1)**	**0 (0)**	**0 (0)**	**0 (0)**	**0 (0)**	**0 (0)**	**0 (0)**	**0 (0)**	**1 (1)**
Traumatic *	1 (1)	0 (0)	0 (0)	0 (0)	0 (0)	0 (0)	0 (0)	0 (0)	1 (1)
**Systemic**	**3 (3)**	**0 (0)**	**2 (2)**	**1 (1)**	**0 (0)**	**0 (0)**	**0 (0)**	**0 (0)**	**0 (0)**
Dehydration	1 (1)	0 (0)	1 (1)	0 (0)	0 (0)	0 (0)	0 (0)	0 (0)	0 (0)
Kwashiorkor *	1 (1)	0 (0)	1 (1)	0 (0)	0 (0)	0 (0)	0 (0)	0 (0)	0 (0)
Severe malnutrition	1 (1)	0 (0)	0 (0)	1 (1)	0 (0)	0 (0)	0 (0)	0 (0)	0 (0)

ǂ Exposed to DTG QD or BID dose with RIF. Numbers exposed to DTG BID: 1 child 3-6kg <6months 10mg DT BID (non-per protocol dose); 3 children 6-<10kg 15DT BID; 2 children 10-<14kg 20DT BID; 1 child 14-<20kg 25DT BID; 2 children 14-<20kg 25FCT BID.

*2 children died: 1 due to Kwashiorkor; 1 due to traumatic accident.

****** 1 event (Hepatitis A) resulted in discontinuation of dolutegravir.

α Follow-up based on time-updated dose. Dose changes were primarily the result of increased weight and moving to the intended PK dose in second PK substudy.

SAEs are analysed as episodes, with all components of the same clinical SAE presented as one episode (e.g. “Thrombocytopenia+Neutropenia”).

For grade ≥3 clinical and laboratory adverse events, each component of the same episode is analysed as a separate event.”

Note: 13 children participated in the first (25mg FCT) and second (25mg DT) pharmacokinetic substudies (14-20Kg) with no overlap; 1 child was exposed to and completed pharmacokinetic profiles on dispersible 15mg while 6-<10kg (12 weeks safety follow-up; 2 grade ≥3 events reported as components of the same clinical SAE) and 20mg while 10-<14kg (24 weeks safety follow-up; no events reported); 1 child was exposed to and completed pharmacokinetic profiles on dispersible 10mg (non per protocol dose; 3 weeks safety follow-up) and 5mg (24 weeks safety follow-up) while 3-<6kg <6months (no events reported); 1 child was exposed to and completed pharmacokinetic profiles on dispersible 20mg FCT (non per protocol dose; 21 weeks safety follow-up) and 25mg DT (24 weeks safety follow-up) while 14-<20kg (no events reported).

SAEs, serious adverse events; **IQR,** interquartile range; **DTG,** dolutegravir; **QD,** once daily; **BID,** twice daily; **DT,** dispersible tablet; **FCT,** film-coated tablet; **<6m**, less than 6 months old; **≥6m**, over 6 months old.

Supplementary table 5. Listing of adverse events reported

ID	Weight	Dose	DTG Exposure (wks)[#]	DTG Dose/Weight Group Exposure (wks)[ǂ]	SAE	Category	Event	Grade	ART Modifying	Relatedness to ART (ERC)
**3**	**3-<6kg**	**5mg DT**	2	2	Yes[1]	Haematological	Thrombocytopenia**^**	Grade 4	No	No
**3**	**3-<6kg**	**5mg DT**	2	2		Haematological	Neutropenia**^**	Grade 4	No	No
**14**	**3-<6kg**	**5mg DT**	11	11	Yes	Infectious Disease	Gastroenteritis	Grade 3	No	No
**2**	**3-<6kg**	**10mg DT**	1	1	Yes	Systemic	Kwashiorkor**	Grade 5	No	No
**13δ**	**3-<6kg**	**10mg DT**	7	7	No	Haematological	Anaemia with no clinical symptoms**^**	Grade 3	No	No
**15**	**3-<6kg**	**10mg DT**	3	1	Yes[3]	Infectious Disease	Acute diarrhoea not investigated	Grade 3	No	No
**15**	**3-<6kg**	**10mg DT**	3	1		Infectious Disease	Dehydration	Grade 3	No	No
**19***	**3-<6kg**	**10mg DT**	1	1	Yes	Infectious Disease	Pneumonia no organism identified	Grade 3	No	No
**19***	**3-<6kg**	**10DT BID**	3	0.1	Yes	Infectious Disease	Tuberculosis - pulmonary - smear negative or not done	Grade 4	No	No
**19***	**3-<6kg**	**10DT BID**	18	15	Yes	Non-HIV related deaths	Traumatic**¥**	Grade 5	No	No
**1**	**6-<10kg**	**15mg DT**	2	2	No	Infectious Disease	Acute diarrhoea not investigated	Grade 3	No	No
**6**	**6-<10kg**	**15mg DT**	10	10	Yes[2]	Systemic	Chest infection	Grade 3	No	No
**6**	**6-<10kg**	**15mg DT**	10	10		Systemic	Severe malnutrition	Grade 4	No	No
**10**	**6-<10kg**	**15mg DT**	3	3	No	Haematological	Anaemia with no clinical symptoms**^**	Grade 3	No	No
**12**	**6-<10kg**	**15mg DT**	8	8	Yes	Infectious Disease	Chronic diarrhoea with no pathogen	Grade 4	No	No
**17**	**6-<10kg**	**15mg DT**	3	3	Yes[4]	Infectious Disease	Acute otitis media	Grade 3	No	No
**17**	**6-<10kg**	**15mg DT**	3	3		Infectious Disease	Pneumonia no organism identified	Grade 3	No	No
**4**	**10-<14kg**	**20mg DT**	2	2	No	Haematological	Anaemia with no clinical symptoms**^**	Grade 3	No	No
**7**	**10-<14kg**	**20mg DT**	22	22	No	Infectious Disease	Raised AST	Grade 3	No	No
**7**	**10-<14kg**	**20mg DT**	22	22	No	Infectious Disease	Hepatitis A	Grade 3	No	No
**11**	**10-<14kg**	**20mg DT**	15	15	No	Biochemical	Raised alkaline phosphatase (ALK)**^**	Grade 3	No	No
**11**	**10-<14kg**	**20mg DT**	14	13	No	Haematological	Neutropenia**^**	Grade 4	No	No
**11**	**10-<14kg**	**20mg DT**	3	3	No	Haematological	Anaemia with no clinical symptoms**^**	Grade 3	No	No
**18**	**10-<14kg**	**20mg DT**	10	10	Yes[5]	Infectious Disease	Measles	Grade 3	No	No
**18**	**10-<14kg**	**20mg DT**	10	10		Infectious Disease	Chest infection	Grade 3	No	No
**8**	**14-<20kg**	**25mg FCT**	19	19	No	Nervous System	Epilepsy, fits, convulsions	Grade 3	No	No
**16**	**14-<20kg**	**25mg FCT**	14	14	Yes	Infectious Disease	Plasmodium falciparum malaria	Grade 3	No	No

**5**	**14-<20kg**	**25mg DT**	24	1	No	Infectious Disease	Hepatitis A	Grade 4	Yes	No
**9[€]**	**14-<20kg**	**25FCT BID**	19	15	Yes	Infectious Disease	Acute febrile episode - undiagnosed	Grade 3	No	No

DTG exposure from randomisation
2 children died: **1 due to Kwashiorkor; ¥1 due to traumatic accident.
ǂ From start of DTG DT following randomisation for <14kg, or start of 25mg FCT (QD or BID) or 25mg DT (QD or BID) for participants participating in first (25mg FCT) and second (25mg DT) pharmacokinetic substudies (14-20Kg)
^ Asymptomatic laboratory event
***** Non-per protocol dose: <6 months 10DT QD received for 3 weeks before increasing to 10DT BID with RIF use.
δ Non-per protocol dose: <6 months 10mg DT
€ 20.1kg and receiving 25FCT BID dosing with RIF use
Note: SAEs are analysed as episodes, with all components of the same clinical SAE presented as one episode.
1 Components of the same clinical SAE (Thrombocytopenia and Neutropenia)
2 Components of the same clinical SAE (Chest infection and Severe malnutrition)
3 Components of the same clinical SAE (Acute diarrhoea not investigated and Dehydration)
4 Components of the same clinical SAE (Pneumonia no organism identified and Acute otitis media)
5 Components of the same clinical SAE (Measles and Chest infection)
SAEs, serious adverse events; **DTG,** dolutegravir; **DT,** dispersible tablet; **FCT,** film-coated tablet; **QD,** once daily; **BID,** twice daily; **ERC,** endpoint review committee.

Supplementary table 6. Association between log AUC_{0-24h} or log C_{max} and occurrence of adverse events

	Odds Ratio*	95% CI	P-value
**Log AUC_{0-24h}**			
Unadjusted	2.0	(0.5, 8.5)	0.327
Adjusted for weight band	3.0	(0.5, 17.0)	0.222
**Log C_{max}**			
Unadjusted	2.7	(0.5, 14.3)	0.245
Adjusted for weight band	5.4	(0.6, 52.8)	0.144

* Odds ratio of adverse event is for each 1-unit increase in log AUC_{0-24h} or log C_{max}. Unadjusted odds ratios are estimated by fitting logistic regression models with the occurrence of adverse event (binary yes/no) as the outcome variable and log AUC_{0-24h} or log C_{max} as the continuous predictor variables. Adjusted models are adjusted for weight band.
Log, natural logarithm; **AUC_{0-24h},** area under the concentration-time curve from 0 to 24 h; **C_{max},** maximum plasma concentration; **95% CI,** 95% confidence interval.

Supplementary figure 3. Log AUC_{0-24h} in children with vs without adverse events reported overall and by weight band

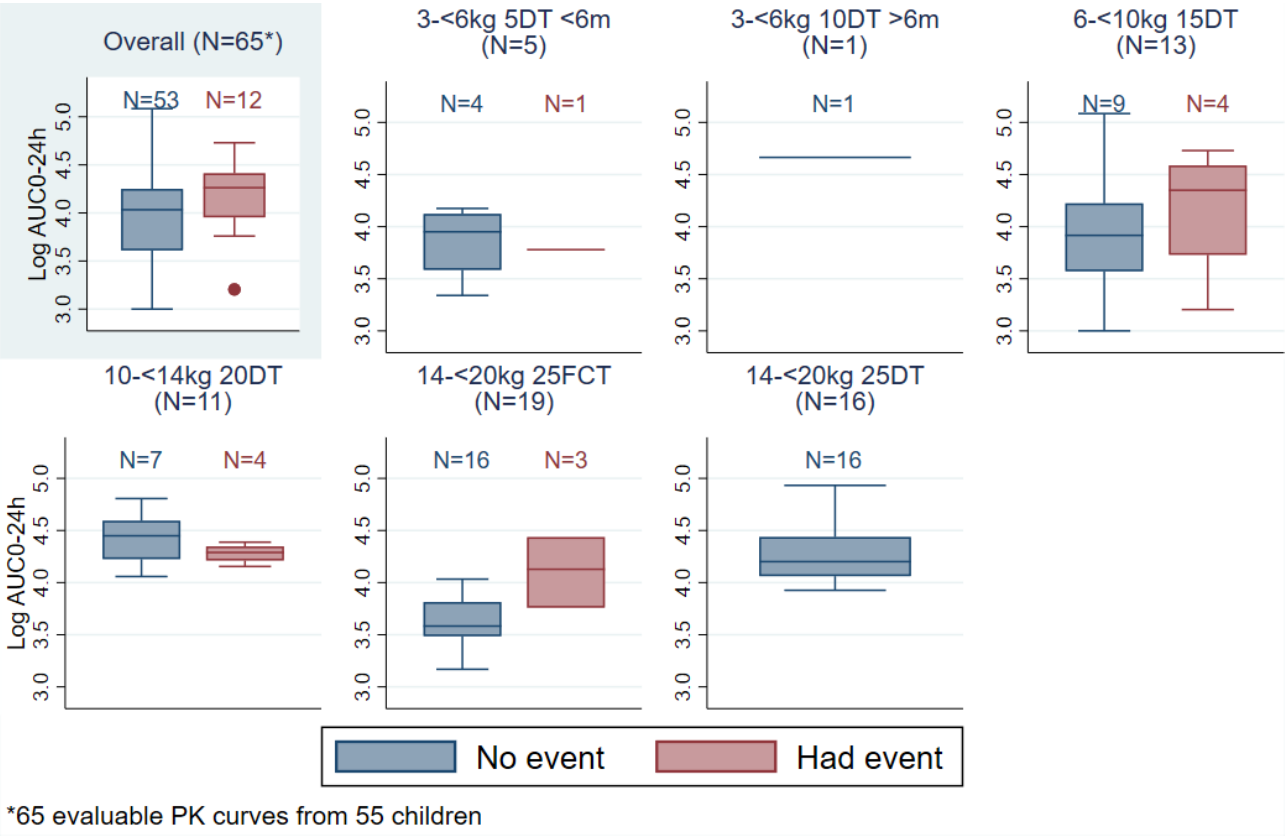

Boxplots are shown. Horizontal lines within the boxes show the median values (Quartile (Q) 2); boxes show interquartile ranges (IQR, Q1 to Q3); upper whiskers denote the uppermost value within Q3 + (IQR × 1·5); lower whiskers denote the lowermost value within Q1 − (IQR × 1·5). **Q1,** 25th percentile; **Q2,** 50th percentile (median); **Q3,** 75th percentile; **DT,** dispersible tablet; **FCT,** film-coated tablet; **Log,** natural logarithm; **AUC_{0-24h},** area under the concentration-time curve from 0 to 24 h; **<6m**, less than 6 months old; **≥6m**, over 6 months old.

Supplementary figure 4. Log C_{max} in children with vs without adverse events reported overall and by weight band

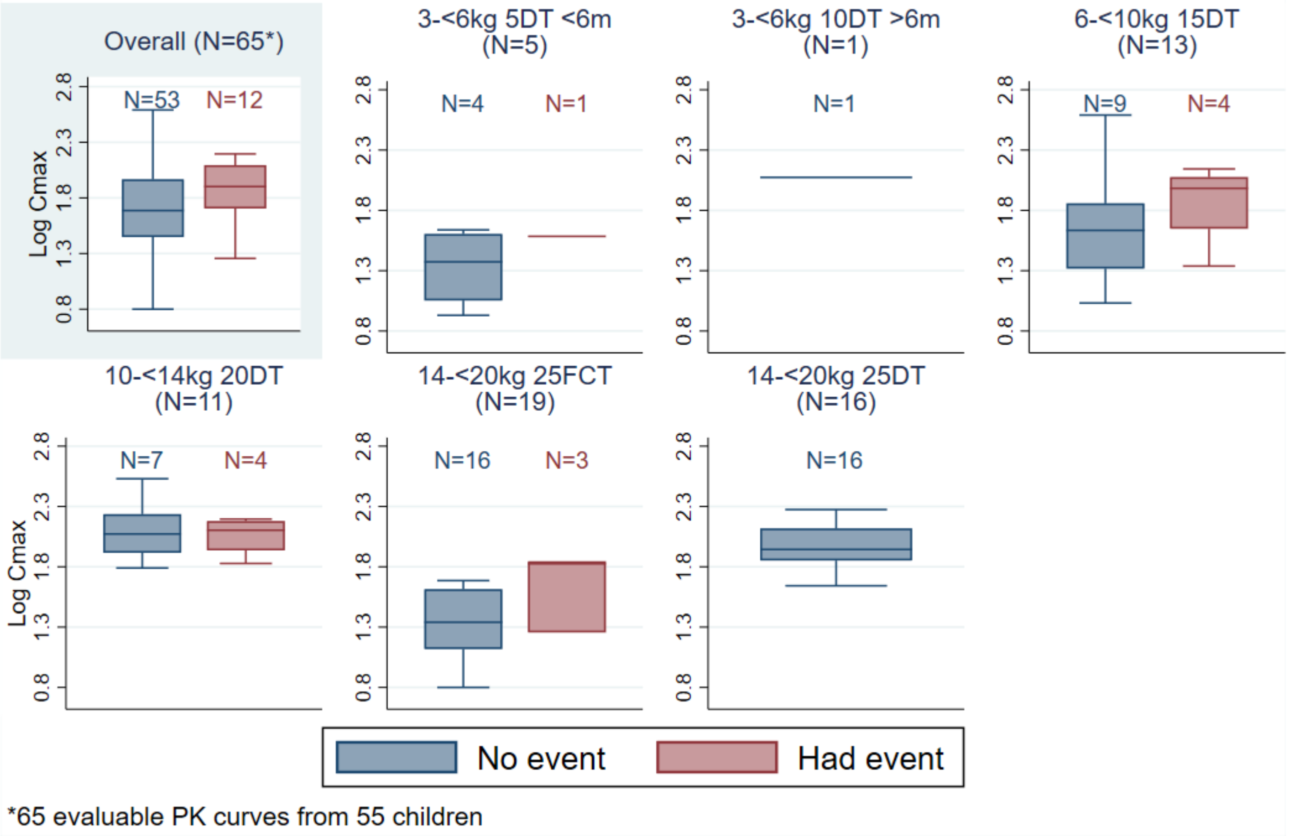

Boxplots are shown. Horizontal lines within the boxes show the median values (Quartile (Q) 2); boxes show interquartile ranges (IQR, Q1 to Q3); upper whiskers denote the uppermost value within Q3 + (IQR × 1·5); lower whiskers denote the lowermost value within Q1 − (IQR × 1·5). **Q1,** 25th percentile; **Q2,** 50th percentile (median); **Q3,** 75th percentile; **DT,** dispersible tablet; **FCT,** film-coated tablet; **Log,** natural logarithm; **C_{max},** maximum plasma concentration; **<6m**, less than 6 months old; **≥6m**, over 6 months old.

Supplementary figure 5. Intra- and inter-subject comparison of pharmacokinetic parameters on 25mg film-coated tablets (FCT; reference) and 25mg dispersible tablets (DT; test)

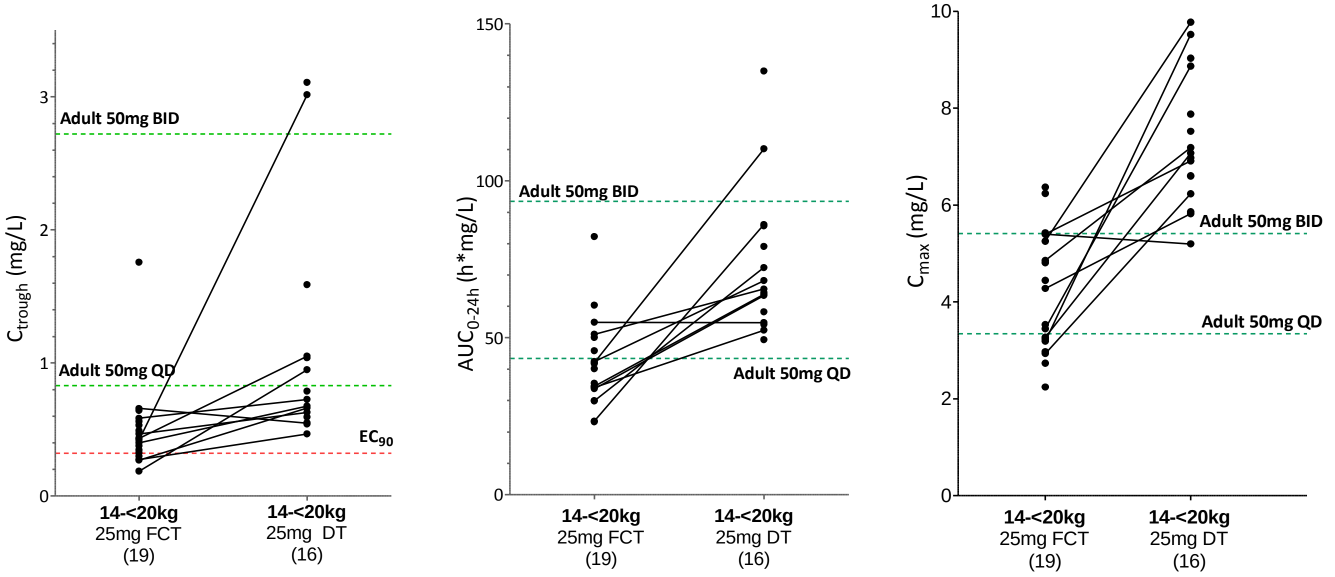

Red line indicates dolutegravir in-vivo EC_{90}. Green dashed lines indicate published geometric mean adult reference values for 50mg once-daily (lower line) and twice-daily doses (upper line).C_{trough}=trough concentration. $AUC_{0\text{–}24h}$=area under the concentration-time curve from 0 to 24 h. C_{max}=maximum concentration. EC_{90}=the effective concentration at which 90% of maximal viral inhibition is achieved in a 10-day monotherapy study.

The ODYSSEY trial team:

ODYSSEY TRIAL TEAM The ODYSSEY Trial Team consists of: (MRC CTU) Shabinah S. Ali, Abdel Babiker, Chiara Borg, Anne-Marie Borges Da Silva, Joanna Calvert, Deborah Ford, Joshua Gasa, Diana M. Gibb, Nasir Jamil, Sarah Lensen, Emma Little, Fatima Mohamed, Samuel Montero, Cecilia L. Moore, Rachel Oguntimehin, Anna Parker, Reena Patel, Tasmin Phillips, Tatiana Sarfati, Karen Scott, Clare Shakeshaft, Moira Spyer, Margaret Thomason, Anna Turkova, Rebecca Turner, Nadine Van Looy, Ellen White, Kaya Widuch, Helen Wilkes, Ben Wynne. (Penta Foundation) Carlo Giaquinto, Tiziana Grossele, Daniel Gomez-Pena, Davide Bilardi, Giulio Vecchia. (INSERM SC-10-US19--ANRS) Alexandra Compagnucci, Yacine Saidi, Yoann Riault, Alexandra Coelho, Laura Picault, Christelle Kouakam. (PHPT) Tim R. Cressey, Suwalai Chalermpantmetagul, Gonzague Jourdain, Nicole Ngo Giang Huong, Dujrudee Chinwong, Chalermpong Saenjum, Rukchanok Peongjakta, Pra-ornsuda Sukrakanchana, Woottichai Khamduang, Laddawan Laomanit, Ampika Kaewbundit, Jiraporn Khamkon, Kanchana Than-in-at, Sanupong Chailert, Worathip Sripaoraya, Nitinart.krueduangkam, Namthip Kruenual, Warunee Khamjakkaew, Soraya Klinprung, (Sub-study Partners) Nigel Klein, Eleni Nastouli, Anita De Rossi, Maria Angeles Munoz Fernandez, Janet Seeley, Sarah Bernays, Stella Namukwaya, Zivai Mupambireyi, Magda Conway, David Burger, Pauline Bollen, Angela Colbers, Hylke Waalewijn. (Joint Clinical Research Centre, Uganda) Cissy M. Kityo, Victor Musiime, Elizabeth Kaudha, Annet Nanduudu, Emmanuel Mujyambere, Paul Ocitti Labeja, Charity Nankunda, Juliet Ategeka, Peter Erim, Collin Makanga, Esther Nambi, Abbas Lugemwa, Lorna Atwine, Edridah Keminyeto, Deogratiuos Tukwasibwe, Shafic Makumbi, Emily Ninsiima, Mercy Tukamushaba, Rogers Ankunda, Ian Natuhurira, Miriam Kasozi, Baker Rubinga, Diana Antonia Rutebarika, Rashida Nazzinda, Shamim Nakabuye, Julius Tumusiime, Alice Mulindwa, Ritah Mbabazi, Milly Ndigendawani, Edward Bagirigomwa, Eddie Rubanga, David Eram, Maria Nannungi, Chrispus Katemba, Disan Mulima, Josephine Namusanje, Mariam Nabalamba, Priscilla Kyobutungi, Phyllis Mwesigwa Rubondo, Robinah Kibenge, Claire Nasaazi, Basiimwa Roy Clark, Enock Babu, Alex Musiime, Faith Mbasani, Martin Ojok, Odoch Denis, David Baliruno, Katabalwa Juliet, Ouma Benson, Barbara Ainebyona. (Baylor College of Medicine Children's Foundation, Uganda) Adeodata R. Kekitiinwa, Pauline Amuge, Dickson Bbuye, Justine Nalubwama, Winnie Akobye, Muzamil Nsibuka Kisekka, Anthony Kirabira, Gloria Ninsiima, Sylvia Namanda, Gerald Agaba, Immaculate Nagawa, Annet Nalugo, Florence Namuli, Rose Kadhuba, Rachael Namuddu, Lameck Kiyimba, Angella Baita, Eunice Atim, Olivia Kobusingye, Clementine Namajja, Africanus Byaruhanga, Rogers Besigye, Herbert Murungi, Geoffrey Onen, Lawrence Lekku, Judith Tikabibamu. (MUJHU Research Collaboration, Uganda) Philippa Musoke, Linda Barlow-Mosha, Grace Ahimbisibwe, Rosemary Namwanje, Hajira Kataike, Mark Ssenyonga, Brenda Kakayi, Rebecca Sakwa, Sarah Nakabuye, Barbara Musoke Nakirya, Gladys Kasangaki, Raymonds Kyambadde, David Balamusani, Winnie Nansamba, Stella Nalusiba, Emmanuel Mayanja, Richard Isabirye, Erinah Kyomukama, Rebecca Wampamba, Mildred Kabasonga, Zaam Zinda Nakawungu, Sarah Babirye, Olivia Kaboggoza, Juliet Nanyonjo, Joanita Nankya Baddokwaya, Alice Elwana, Winfred Kaahwa, Bosco Kafufu, Emmanuel Hakiza, Maria Musisi, Paula Namayanja, Maria Gorreti Nakalema, Robert Serunjogi, Monica Etima, Phionah Kibalama, Joel Maena, Agnes Mary Mugagga, Annet Miwanda, Monica Nolan. (FAM-CRU, South AfricaMark F. Cotton, Anita Janse van Rensburg, Marlize Smuts, Catherine Andrea, Sumaya Dadan, Sonja Pieterse, Vinesh Jeaven, Candice Makola, George Fourie, Kurt Smith, Els Dobbels, Peter Zuidewind, Hesti Van Huyssteen, Mornay Isaacs, Georgina Nentsa, Thabisa Ncgaba, Candice MacDonald, Maria Bester, Wilma Orange, Ronelle Arendze, Mark Mulder, Lucille Malgraaf, Ashley Harley. (PHRU, South Africa) Avy Violari, Nastassja Ramsagar, Afaaf Liberty, Ruth Mathiba, Mandisa Nyati, Haseena Cassim, Lindiwe Maseko, Nkata Kekane, Busi Khumalo, Mirriam Khunene, Noshalaza Sbisi, Jackie Brown, Tryphina Madonsela, Nokuthula Mbadaliga, Zaakirah Essack, Reshma Lakha, Aasia Vadee, Derusha Frank, Nazim Akoojee, Maletsatsi Monametsi, Gladness Machache, Yolandie Fourie, Anusha Nanan-kanjee, Juan Erasmus, Angelous Mamiane, Tseleng Daniel, Fatima Mayat, Nomfundo Maduna, Patsy Baliram, Sibongile Sithebe, Emily Lebotsa, Siphiwe Mkhize.. (Prapokklao Hospital, Thailand) Chaiwat Ngampiyaskul, Pisut Greetanukroh, Praechadaporn Khannak, Pathanee Tearsansern, Wanna Chamjamrat. (Phayao Hospital, Thailand) Nuttawat Chanto, Thitiwat Thapwai, Khanungnit Thungkham, Patcharee Puangmalai, Chutima Ruklao. (Chiangrai Prachanukroh Hospital, Thailand) Pradthana Ounchanum, Suwimon Khusuwan, Sukanda Denjanta, Yupawan Thaweesombat, Jutarat Thewsoongnoen, Kanyanee Kaewmamueng, Phakamas Kamboua, Supawadee Pongprapass (Sangjan), Warunee Srisuk, Areerat Kongponoi, Juthamas Limplertjareanwanich. (Nakornping Hospital, Thailand) Suparat Kanjanavanit, Prattana Leenasirimakul, Chayakorn Saewtrakool,

Pacharaporn Yingyong, Duangrat Chutima (Suwan), Rangwit Junkaew, Orapin Khatngam, Thannapat Chankun. (Khon Kaen Hospital, Thailand) Ussanee Srirompotong, Patamawadee Sudsaard, Sookpanee Wimonklang, Turian Petpranee.. (Mahasarakam Hospital, Thailand) Sathaporn Na-Rajsima, Pattira Runarassamee, Nuananong Kunjaroenrut, Arttasid Udomvised, Tassawan Khayanchoomnoom, Watchara Meethaisong, Ketmookda Trairat.. (HIVNAT, Thailand) Thanyawee Puthanakit, Suvaporn Anugulruengkitt, Wipaporn Natalie Songtaweesin, Torsak Bunupuradah, Naruporn Kasipong, Sararut Chanthaburanun, Apicha Mahanontharit, Kesdao Nanthapisal, Thidarat Jupimai, Thornthun Noppakaorattanamanee, Chutima Saisaengjan.. (Klerksdorp Tshepong Hospital Complex, South Africa) Ebrahim Variava, Modiehi Rakgokong, Dihedile Scheppers, Tumelo Moloantoa, Abdul Hamid Kaka, Tshepiso Masienyane, Akshmi Ori, Kgosimang Mmolawa, Pattamukkil Abraham . (Durban International Clinical Research Site, South Africa) Moherndran Archary, Rosie Mngqibisa, Rejoice Mosia, Sajeeda Mawlana, Rashina Nundlal, Penelope Madlala, Allemah Naidoo, Sphiwee Cebekhulu, Petronelle Casey, Subashinie Sidhoo, Minenhle Chikowore, Lungile Nyantsa, Sheleika Singh . (AHRI, South Africa) Nigel Klein, Osee Behuhuma, Kristien Bird, Olivier Koole, Nomzamo Buthelezi, Siva Danaviah, Theresa Smit, Gugulethu Gasa, Mumsy Mthethwa.. (UZCRC, Zimbabwe) James Hakim, Hilda Mujuru, Kusum Nathoo, Mutsa Bwakura-Dangarembizi, Ennie Chidziva, Shepherd Mudzingwa, Secrecy Gondo, Godfrey Musoro, Vivian Mumbiro, Gloria Tinago, Shirley Mutsai, Joy Chimanzi, Columbus Moyo, Ruth Nhema, Misheck Nkalo Phiri, Stuart Chitongo, Joshua Choga, Joyline Bhiri, Wilber Ishemunyoro, Makhosonke Ndlovu, Moses Chitsamatanga, Pia Ngwaru, Tsitsi Gwenzi, Wendy Mapfumo, Dorothy Murungu, Trust Mukanganiki, Prosper Dube, Tapiwa Gwaze, Farai Matimba, Tawona Mudzviti, Zivai Mupambireyi, Sibusisiwe Weza, Cleopatra Langa, Sandra Musarurwa, Shamiso Gwande.. (Goethe University Frankfurt, Germany) Stephan Schultze-Straber, Christoph Konigs. (UKE Eppendorf, Germany) Robin Kobbe, Ulf Schulze-Sturm, Felicia Mantkowski, Cornelius Rau. (Heartlands Hospital, UK) Steve Welch, Jacqui Daglish, Laura Thrasyvoulou, Kate Gandhi, Yvonne Vaughan-Gordon (Great Ormand Street Hospital, UK) Delane Singadia, Sophie Foxall, Judith Acero, Gosia Pasko-Szcech, Jacquie Flynn. (St Mary's Hospital, UK) Gareth Tudor-Williams, Farhana Abdulla, Caroline Foster, Sobia Mustafa. (Leicester Royal Infirmary, UK) Srini Bandi, Jin Li, Jackie Philps. (Leeds General Infirmary, UK) Sean O'Riordan, Dominique Barker, Richard Vowden, Maria Dowie. (Kings College Hospital, UK) Colin Ball Eniola Nsirim, Kathleen McClaughlin. (Hospital 12 de Octubre, Spain) India Garcia, Pablo Rojo Conejo, Cristina Epalza, Luis Prieto Tato, Maite Fernandez, Luis Escosa Garcia. (Hospital La Paz, Spain) Maria José Mellado Peña, Talia Sainz Costa. (Hospital San Joan de Déu, Spain) Claudia Fortuny Guasch, Antoni Noguera Julian, Carolina Estepa, Elena Bruno, Patricia Mendez Garcia, Alba Murciano Cabeza, Biobanco Gregorio Maranon, Maria Angeles Muñoz Fernandez, Jose Luis Jimenez, Coral Gomez Rico. (Centro Materno-infantile do Norte, Portugal) Laura Marques, Carla Teixeira, Alexandre Fernandes, Rosita Nunes, Helena Nascimento, Andreia Padrao, Joana Tuna, Helena Ramos, Ana Constança Mendes, Helena Pinheiro, Ana Cristina Matos. (Local Site Monitors) Flavia Kyomuhendo, Sarah Nakalanzi, Cynthia Mukisa Williams, Leora Sewnarain, Ntombenhle Ngcobo, Deborah Pako, Nompumelelo Yende, Jacky Crisp, Marlize Smuts, Benedictor Dube, Precious Chandiwana, Winnie Gozhora, Thidarat Jumpimai. (Independent Trial Steering Committee Members) Ian Weller, Elaine Abrams, Tsitsi Apollo, Polly Clayden, Valériane Leroy. (Independent Data Monitoring Committee Members) Anton Pozniak, Jane Crawley, Rodolphe Thiébaut, Helen McIlleron. (Endpoint Review Committee Members) Alasdair Bamford, Hermione Lyall, Andrew Prendergast, Felicity Fitzgerald, Anna Goodman
